# Deficiency of muscular dystrophy–related gene *JAG2* causes NOTCH signaling dysfunction in muscle stem cells

**DOI:** 10.1172/JCI198639

**Published:** 2026-05-19

**Authors:** Minoru Tanaka, Nam Chul Kim, Isabelle Draper, Hannah R. Littel, Mekala Gunasekaran, Johnnie Turner, Natalya M. Wells, Qasim Mujteba, Yoko Asakura, Peter B. Kang, Atsushi Asakura

**Affiliations:** 1 Stem Cell Institute and; 2 Greg Marzolf Jr. Muscular Dystrophy Center and Department of Neurology, University of Minnesota Medical School, Minneapolis, Minnesota, USA.; 3 Department of Pharmacy Practice and Pharmaceutical Sciences, University of Minnesota College of Pharmacy, Duluth, Minnesota, USA.; 4 Molecular Cardiology Research Institute, Tufts Medical Center, Boston, Massachusetts, USA.; 5 Institute for Translational Neuroscience, University of Minnesota Medical School, Minneapolis, Minnesota, USA.

**Keywords:** Development, Muscle biology, Adult stem cells, Mouse models, Neuromuscular disease

## Abstract

We previously identified a muscular dystrophy caused by biallelic variants in *JAGGED2* (*JAG2*), whose protein product, JAG2, is a canonical NOTCH ligand. However, the disease mechanism remains unclear, particularly with respect to muscle stem cell (muscle satellite cell/MuSC) function and muscle regeneration. We examined the consequences of *JAG2* deficiency and modeled pathogenic *JAG2* variants in vitro and in vivo, the latter in mouse and fly models and with particular attention to the MuSC–muscle endothelial cell (MuEC) niche. We found that both *Jag2* deficiency and overexpression of pathogenic *JAG2* variants impaired NOTCH signaling and myogenic self-renewal and differentiation. Hypomorphic *Jag2* mutant (*Jag2^sm^*) mice displayed depleted MuSCs, corresponding with impaired muscle regeneration in those mice. Coculture experiments and the examination of cell type–specific *Jag2* conditional knockout mice demonstrated that MuEC-specific *Jag2* knockout resulted in reduced MuSC self-renewal, while MuSC-specific *Jag2* knockout resulted in reduced myogenic differentiation. Human reference *JAG2*, but not human pathogenic variants of *JAG2*, rescued the deficiency of *Serrate*, the *Drosophila* ortholog of *JAG2*. Therefore, pathogenic variants in *JAG2* impair muscle development and regeneration through disrupted cell-autonomous *cis-*inhibition and nonautonomous *trans-*activation involving NOTCH signaling dysfunction. Our findings indicate that optimizing JAG2-mediated NOTCH signaling is a potential therapeutic approach for *JAG2*-related muscular dystrophy.

## Abstract 2

A muscular dystrophy caused by biallelic variants in *JAG2*. *JAG2* mutations impair muscle regeneration through disrupted cell-autonomous cis-inhibition and non-autonomous trans-activation involving Notch signaling dysfunction.

## Graphical abstract

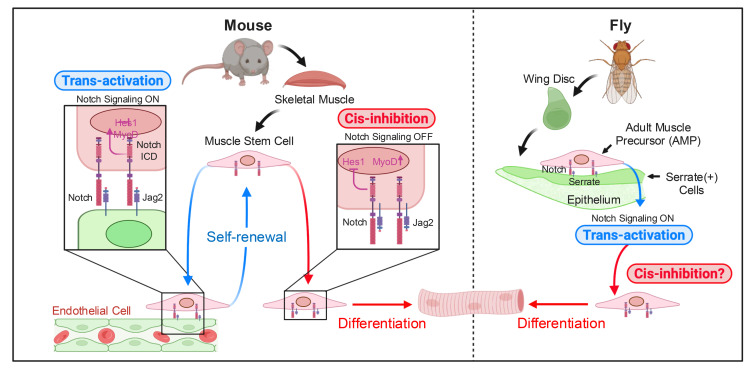

## Introduction

Muscle stem cells (MuSCs), also known as satellite cells, are normally quiescent cells located underneath the basal lamina of muscle fibers. In response to injury, MuSCs activate, proliferate, differentiate, and either form new muscle fibers or fuse with existing muscle fibers to repair the damaged muscle. A small proportion of activated MuSCs self-renew or escape activation to replenish the MuSC pool (1). Defects in self-renewal lead to fewer MuSCs and diminished muscle regenerative capacity, particularly in aged and diseased muscle (1, 2). Prior studies have elucidated the molecular mechanisms regulating MuSCs through the NOTCH, WNT, FGF, and extracellular matrix signaling pathways, as well as juxtacrine interactions (3). We and others have studied the role of muscle endothelial cells (MuECs) in regulating MuSCs (4–6). We have shown that increasing vascular density can augment MuSC numbers (7–9), which is mediated through the activation of NOTCH signaling in MuSCs by DLL4, a MuEC-derived NOTCH ligand (6).

In mammalian cells, NOTCH signaling involves the transmembrane NOTCH ligands JAG1, JAG2, DLL1, DLL3, and DLL4, which bind to NOTCH receptors 1–4 (10), and has been implicated in the homeostasis of stem cells, including MuSCs. NOTCH signaling regulates the maintenance of MuSC quiescence (3, 11, 12), as well as the formation of the MuSC niche (13). However, the precise mechanisms of NOTCH ligand contributions to niche formation and NOTCH activation in MuSCs remain unclear. Endothelial cells express NOTCH ligands, which in turn regulate stem cells (14), including hematopoietic stem cells (15–18), neural stem cells (19), and MuSCs (6).

Disruption of NOTCH signaling is associated with several skeletal muscle diseases, particularly those associated with *JAG2* (20), *MEGF10* (21, 22), *POGLUT1* (23), and *NOTCH2NLC* (10). We discovered that biallelic pathogenic variants in *JAG2* are associated with congenital muscular dystrophy and limb-girdle muscular dystrophy (20). Human JAG2 is a 1,238–amino acid membrane protein that interacts with NOTCH receptors via extracellular EGF domains (24), triggering cell–cell interaction–mediated *trans-*activation of NOTCH signaling (10). JAG2 binding to a NOTCH receptor leads to double cleavage, followed by migration of the NOTCH intracellular domain to the nucleus, where it regulates transcription (25).

The orthologous *Serrate* (*Ser*) gene in *Drosophila* was identified in 1990 (26), followed by *Jag2* in mammals (27, 28). JAG2 is expressed in mammalian skeletal muscle, MuECs, and MuSCs (6, 29).

NOTCH ligands suppress or activate NOTCH signaling cell-autonomously through *cis-*inhibition or via *trans-*activation of NOTCH receptors on neighboring cells (30–34). The exact ligand-receptor mechanisms that regulate NOTCH signaling in MuSCs remain unclear. For this study, we examined the *cis-* and *trans-*regulatory activities of JAG2 for MuSC function and skeletal muscle regeneration in *Jag2* hypomorphic (*Jag2^sm^*) mice, as well as in conditional MuEC- and MuSC-specific *Jag2*-KO mice. We utilized MuEC-MuSC coculture systems to examine JAG2-mediated *trans-*activities of NOTCH for MuSCs. Lastly, we introduced reference and variant forms of human *JAG2* into MuSC cultures and *Drosophila*.

## Results

### Jag2 expression patterns in MuSCs and MuECs.

**Figure 1:**
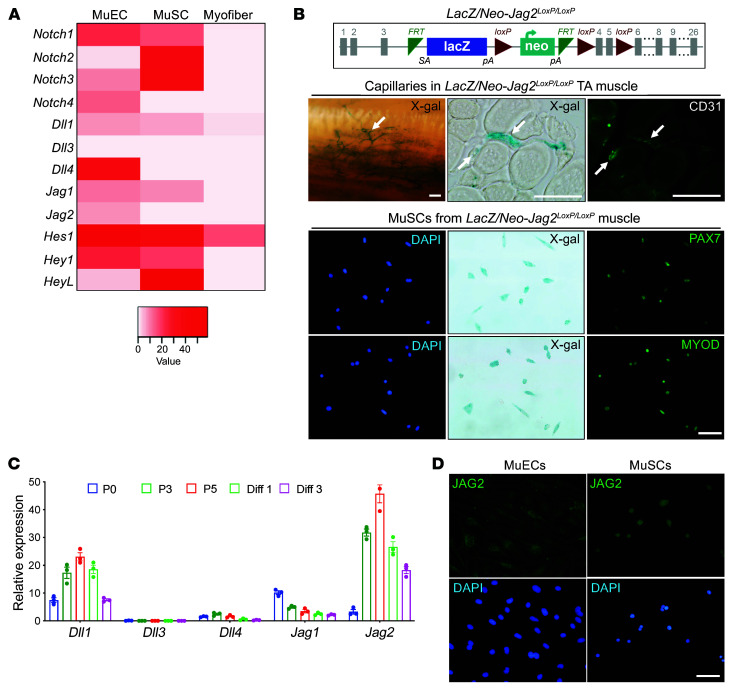
*Jag2* is expressed in MuSCs and MuECs. (**A**) Heatmap for gene expression from our published RNA-seq data (GEO, GSE108739) reveals active NOTCH signaling in freshly isolated MuECs and MuSCs, but not in muscle fibers (14). Heatmap was created via Heatmapper software (https://heatmapper2.ca). (**B**) Upper panel: schematic genomic structure of *Jag2^LoxP/LoxP^* locus for conditional *Jag2* mutant (*LacZ/Neo-Jag2^LoxP/LoxP^*) mice before Flippase-mediated recombination. *LacZ* gene cassette was inserted in intron 3 of the *Jag2* locus, allowing us to detect LacZ^+^ *Jag2*-expressing cells following X-gal staining. Middle panels: on X-gal staining, capillaries (white arrows) in whole TA muscle from *LacZ/Neo-Jag2^LoxP/LoxP^* mice are positive for LacZ^+^, and the muscle sections show that LacZ^+^ cells express CD31 (arrows). Lower panels: isolated MuSCs from *LacZ/Neo-Jag2^LoxP/LoxP^* mice are positive for LacZ^+^ and PAX7 or MYOD. (**C**) RT-qPCR shows upregulation and downregulation of *Dll1* and *Jag2* following MuSC activation and differentiation, respectively. P0, P3, P5, Diff 1, and Diff 3 denote freshly isolated MuSCs, passage day 3, passage day 5, differentiation day 1, and differentiation day 5. Data are shown as the mean ± SEM. (**D**) Anti-JAG2 antibody staining shows JAG2 expression at the membrane of MuECs and MuSCs. DAPI stained all nuclei (blue). Scale bars: 50 μm. Data are shown as the mean ± SEM; *n* = 3 biological replicates.

NOTCH signaling is important for cell communication in skeletal muscle (6, 35, 36). Previously, we performed a directional interactome analysis with MuECs as the sending cells and MuSCs as the receiving cells. Gene Ontology (GO) term–mediated interactome analysis identified NOTCH signaling–mediated interactions between MuECs and MuSCs, including MuEC-derived *Jag2* and *Dll4* interacting with MuSC-derived *Notch2* and *Notch3* (6). We verified expression of NOTCH signaling pathway genes via RNA-seq (Figure 1A). Freshly isolated MuECs express the NOTCH ligands *Dll1*, *Dll4*, *Jag1*, and *Jag2*. Freshly isolated MuSCs express the NOTCH receptors *Notch1*, *Notch2*, and *Notch3*, as well as the NOTCH ligands *Dll1* and *Jag1*, with lower expression levels of *Dll4* and *Jag2*. High expression of the NOTCH downstream genes *Hes1*, *Hey1*, and *Heyl* indicates that NOTCH signaling maintains MuSCs. We confirmed *Jag2* expression in MuECs and MuSCs using a *Jag2^LoxP/LoxP^* mouse line with a *LacZ/Neo* cassette that enabled us to detect *Jag2*-expressing cells in muscle. X-gal staining of whole tibialis anterior (TA) muscle and purified MuSCs from *LacZ/Neo*-*Jag2^LoxP/LoxP^* mice revealed high levels of β-galactosidase activity in CD31^+^ capillaries but low levels in PAX7^+^ and MYOD^+^ MuSCs (Figure 1B). RT-qPCR showed that *Jag2* expression is low in quiescent MuSCs, upregulated during activation, and downregulated during myogenic differentiation (Figure 1C). JAG2 is detected in the membranes of cultured MuECs and MuSCs (Figure 1D). We concluded that JAG2 is expressed in MuECs and variably expressed in MuSCs, depending on their stage in myogenesis.

### Downregulation of NOTCH signaling and myogenic differentiation genes in Jag2 deficiency.

To understand the influence of JAG2 on NOTCH signaling during myogenesis, we measured the gene expression levels of 93 NOTCH- and myogenesis-related genes (Supplemental Table 1A; supplemental material available online with this article; https://doi.org/10.1172/JCI198639DS1) via qPCR in C2C12 myoblasts treated with *Jag2* shRNA compared with scrambled shRNA controls cultured in differentiation media for 7 days. In *Jag2* shRNA–treated cells, 23 genes were significantly downregulated, notably *Jag1*, *Jag2*, *Megf10*, *Notch1*, *Notch3*, and *MyoG* (Supplemental Figure 1A and Supplemental Table 1). No genes were significantly upregulated. After 7 days of differentiation, *Jag2* and *MyoG* expression levels were lower in *Jag2* shRNA–treated cells (Supplemental Figure 1, B and C), with a lower myotube fusion index (Supplemental Figure 1D). Phase contrast analysis showed reduced multinucleated myotube formation in *Jag2* shRNA–treated cells (Supplemental Figure 1E). Similarly, WT MuSCs transfected with *Jag2* siRNA showed significantly reduced myosin heavy chain (MyHC^+^) differentiating cells but not MYOD compared with control siRNA on day 1 of differentiation conditions (Supplemental Figure 1, F–H). These data suggest that JAG2 regulates myogenic differentiation by activating NOTCH signaling.

### Reduced MuSCs in homozygous Jag2^sm^ mice.

**Figure 2:**
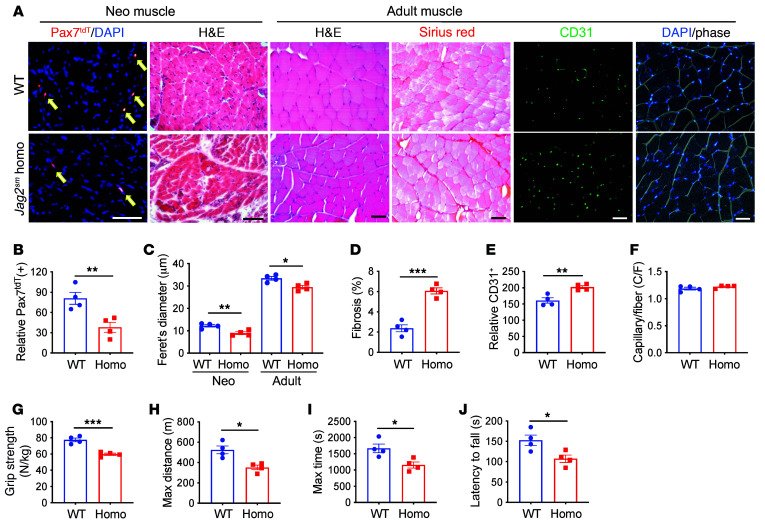
Muscle phenotypes in *Jag2^sm^* mice. (**A**–**F**) Muscle from 4-day-old and 3-month-old *Jag2^sm^*Homo *Pax7^CreERT2^ R26R^tdTomato^* mice showed reduced Pax7^tdTomato^ (Pax7^tdT+^) MuSCs (arrows) in neonatal TA muscle following TMX injection prior to euthanasia (**B**), reduced fiber diameters in both neonatal and adult TA muscle (**C**), increased Sirius red^+^ fibrosis in adult TA muscle (**D**), and increased CD31^+^ capillary density (**E**) but no alteration for capillary per fiber ratio (C/F) (**F**) in adult TA muscle versus *Jag2^sm^*WT *Pax7^CreERT2^ R26^tdTomato^* mice. (**G**) Grip strength is reduced in *Jag2^sm^* homozygous versus WT mice. (**H** and **I**) Treadmill running time and distance are reduced in *Jag2^sm^* homozygous versus WT mice. (**J**) Motor coordination or balance on the rotarod was impaired in *Jag2^sm^* homozygous versus WT mice. DAPI stained all nuclei (blue). Scale bars: 100 μm. Unpaired 2-tailed Student’s *t* tests; *P < 0.05, **P < 0.01, and ***P < 0.001. Data are shown as the mean ± SEM; *n* = 4 biological replicates.

*Jag2^sm^* mice harbor a naturally occurring *p.Gly267Ser* variant in the first EGF repeat in the extracellular domain, which is important for NOTCH signaling (37). Although the homozygous *Jag2^sm^* mouse displays syndactyly and cleft palate (38), a skeletal muscle phenotype has not been previously described. At 4 days after birth, PAX7^+^ MuSCs were reduced in homozygous *Jag2^sm^* mice (Figure 2, A and B). The TA muscle of neonatal homozygous *Jag2^sm^* mice had smaller Feret’s fiber diameters (Figure 2, A and C). Adult homozygous *Jag2^sm^* mice showed reduced body mass and muscle weight (Supplemental Figure 2). The muscle defects in neonatal homozygous *Jag2^sm^* mice persisted in adult TA muscle with reduced Feret’s fiber diameters and increased fibrosis (Figure 2, A, C, and D). While CD31^+^ capillary density was increased in the TA muscle of adult homozygous *Jag2^sm^* mice (Figure 2, A and E), the ratio of capillary per fiber was not changed between WT and homozygous mice (Figure 2F). Homozygous *Jag2^sm^* mice showed reduced forelimb muscle grip strength (Figure 2G), running durations and distances (Figure 2, H and I), and rotarod running time (Figure 2J). Homozygous *Jag2^sm^* mice exhibited impaired MuSC development, leading to impaired skeletal muscle development and function.

**Figure 3:**
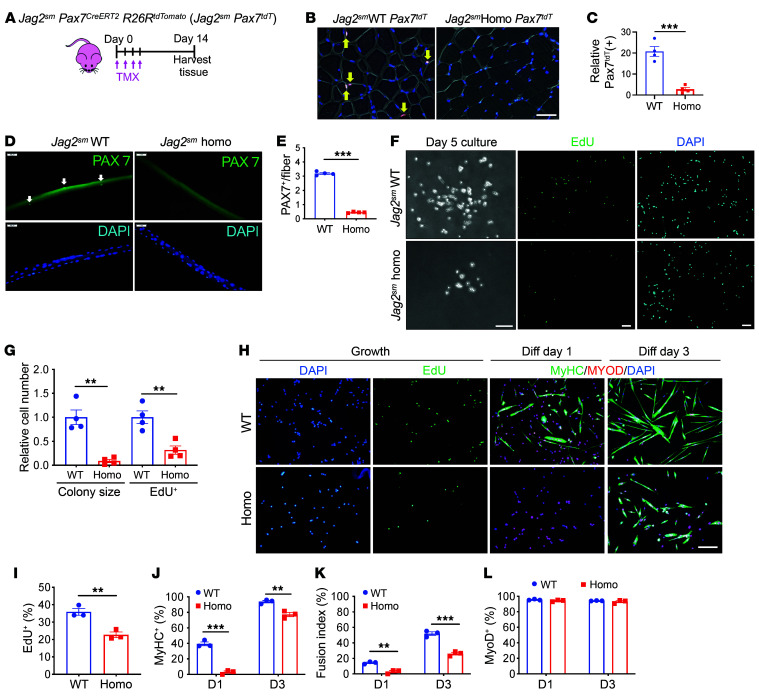
MuSCs are depleted in *Jag2^sm^* homozygous mice. (**A**) *Jag2^sm^* *Pax7^CreERT2^ R26R^tdTomato^* (*Jag2^sm^ Pax7^tdT^*) mice were injected with TMX prior to euthanasia. (**B**–**E**) TA muscle sections (**B** and **C**) and isolated single-muscle fibers (**D** and **E**) demonstrated reduced Pax7^tdTomato^ (Pax7^tdT+^) MuSCs (arrows, pink and green colors, respectively) in *Jag2^sm^* homozygous versus WT mice. (**F** and **G**) Freshly isolated MuSCs (5 × 10^4^ cells/3 cm plate) from WT and homozygous *Jag2^sm^* mice were seeded, and *Jag2^sm^* homozygous MuSCs show reduced colony sizes and EdU^+^ proliferating cells. (**H**–**L**) MuSCs isolated from homozygous *Jag2^sm^* mice (**H**) show reduced EdU^+^ proliferating cells (**I**; green) and MyHC^+^ myogenic differentiation (green) and fusion in days 1 and 3 differentiation conditions (**J** and **K**), while MYOD^+^ cells (**L**; red) are not altered versus WT cells. DAPI stained all nuclei (blue). Scale bars: 20 μm (**B**), 50 μm (**D**), 100 μm (**F** and **H**). Unpaired 2-tailed Student’s *t* tests; **P < 0.01 and ***P < 0.001. Data are shown as the mean ± SEM; *n* = 4 (**D** and **E**) and *n* = 3 (**I**–**L**) biological replicates.

We crossed homozygous *Jag2^sm^* mice with *Pax7^+/CreERT2^ ROSO26^+/Loxp-stop-Loxp-tdTomato^* (*Pax7^CreERT2^ R26R^tdT^* or *Pax7^tdT^*) mice to generate *Jag2^sm^ Pax7^tdT^* mice. The control mice (*Jag2^WT^ Pax7^tdT^*) mark MuSCs as tdTomato^+^ cells (39). We confirmed that tdTomato was specifically expressed in the cells of interest after tamoxifen (TMX) injection (Figure 3, A and B). Significantly fewer MuSCs were detected in TA muscle cross sections from adult *Jag2^sm^ Pax7^tdT^* mice (Figure 3, B and C). Single-muscle fiber analysis isolated from adult mice also showed significantly reduced PAX7^+^ MuSC counts in *Jag2^sm^* extensor digitorum longus muscle fibers compared with control mice (Figure 3, D and E).

### Reduced cell proliferation and myogenic differentiation of MuSCs isolated from homozygous Jag2^sm^ mice.

MuSCs were isolated from hind limb muscles of adult *Jag2^sm^* mice using antibody-mediated magnetic sorting. Freshly isolated MuSCs were cultured in growth medium to assess proliferation for 5 days. *Jag2^sm^* MuSCs displayed reduced cell proliferation, as evidenced by smaller colony sizes (Figure 3, F and G). A 5-ethynyl-2′-deoxyuridine (EdU) incorporation assay correspondingly revealed reduced proliferating cells. We assessed the cell proliferation of passaged MuSCs, then switched to differentiation medium for 1 or 3 days to evaluate myogenic differentiation (Figure 3H). There were reduced proliferating (EdU^+^) MuSCs in *Jag2^sm^* (Figure 3, H and I). Annexin V and TUNEL staining showed that apoptotic cell death of *Jag2^sm^* MuSCs was slightly increased compared with WT MuSCs following thapsigargin or UV treatment (Supplemental Figure 3). These findings confirm Jag2’s essential role in niche-independent MuSC proliferation and survival. During myogenic differentiation, the number of MYOD^+^-committed myogenic progenitors remained unchanged in homozygous *Jag2^sm^* mice (Figure 3, H and L). However, immunostaining for MyHC after 1 or 3 days of differentiation showed diminished multinucleated myotubes in *Jag2^sm^* MuSC cultures (Figure 3, H, J, and K). These findings suggest that while *Jag2* deficiency does not affect the initial commitment of MuSCs to the MYOD^+^ myogenic lineage, it impairs their proliferative capacity and subsequent myogenic differentiation.

### Impaired muscle regeneration in homozygous Jag2^sm^ mice.

**Figure 4:**
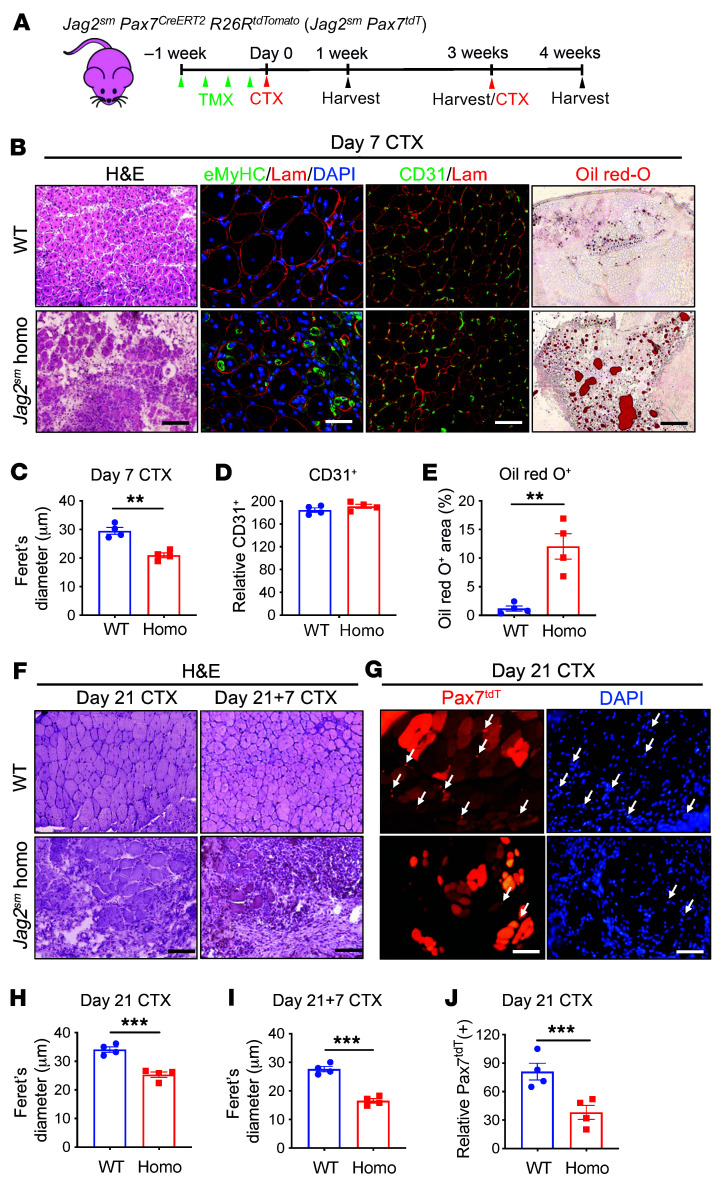
*Jag2^sm^* hypomorphic mice have muscle regenerative defects. (**A**) Single or repeated CTX injections into the TA muscle were performed on *Jag2^sm^*WT *Pax7^CreERT2^ R26^tdTomato^* and *Jag2^sm^*homo *Pax7^CreERT2^ R26^tdTomato^* mice following TMX injection. (**B**) TA histology (H&E and Oil Red O) and immunostaining (eMyHC/Laminin/DAPI and CD31/Laminin) 7 days following CTX injection into the TA. Scale bars from left to right: 100, 25, 50, and 250 μm. (**C**) Feret’s diameters of TA fibers in WT and *Jag2^sm^* homozygous mice at 7 days following CTX injection. (**D**) CD31^+^ capillaries in TA muscle in WT and *Jag2^sm^* homozygous mice 7 days following CTX injections. (**E**) Oil Red O^+^ area was evaluated at 7 days following CTX injection. (**F**) H&E staining at 21 days and 21+7 days following CTX injections into the TA. (**G**) Pax7^tdTomato^ (Pax7^tdT+^) MuSCs (arrows) at 21 days following CTX injections into the TA. Scale bars: 100 μm. (**H** and **I**) Feret’s diameters of TA fibers in WT and *Jag2^sm^* homozygous mice at 21 days and 21+7 days following CTX injection. (**J**) Pax7^tdT+^ MuSCs in WT and *Jag2^sm^* homozygous at 21 days following CTX injections into TA muscle. DAPI stained all nuclei (blue). *n* = 4 independent experiments. Unpaired 2-tailed Student’s *t* tests; **P < 0.01 and ***P < 0.001. Data are shown as the mean ± SEM; *n* = 4 biological replicates.

We assessed the regenerative capacity of TA muscles in adult *Jag2^sm^ Pax7^tdT^* mice following TMX injection and intramuscular cardiotoxin-induced (CTX-induced) injury (Figure 4A). At day 7 after injury, *Jag2^sm^ Pax7^tdT^* TA showed smaller regenerating muscle fibers (Figure 4B). Feret’s muscle fiber diameters were reduced in *Jag2^sm^ Pax7^tdT^* TA (Figure 4C) at 7 days after injury. Immunostaining showed that embryonic MyHC (eMyHC), a marker of newly formed fibers, persisted in *Jag2^sm^* muscle at day 7 after injury but was no longer detectable in regenerating WT muscle (Figure 4B), indicating delayed muscle regeneration in *Jag2* deficiency. No differences were observed in the number of CD31^+^ capillaries between homozygous and WT muscle (Figure 4, B and D), indicating that the muscle regenerative defects are unlikely to be related to differences in muscle microvasculature. Oil Red O staining revealed increased lipid accumulation in regenerating *Jag2^sm^* muscle (Figure 4, B and E). At 21 and 21+7 days following sequential CTX injections in *Jag2^sm^ Pax7^tdT^* mice, muscle regeneration was significantly impaired (Figure 4, F–I), suggesting defects in MuSC self-renewal. Supporting this, both number of Pax7^tdT+^ MuSCs and Pax7^tdT+^ MuSC-derived regenerating muscle fibers were significantly reduced in *Jag2^sm^ Pax7^tdT^* mice at 21 days following CTX injections (Figure 4, G and J), strongly suggesting the reduction of MuSCs and muscle regeneration in *Jag2* deficiency.

### Transcriptome sequencing (RNA-seq) of homozygous Jag2^sm^ MuSCs.

**Figure 5:**
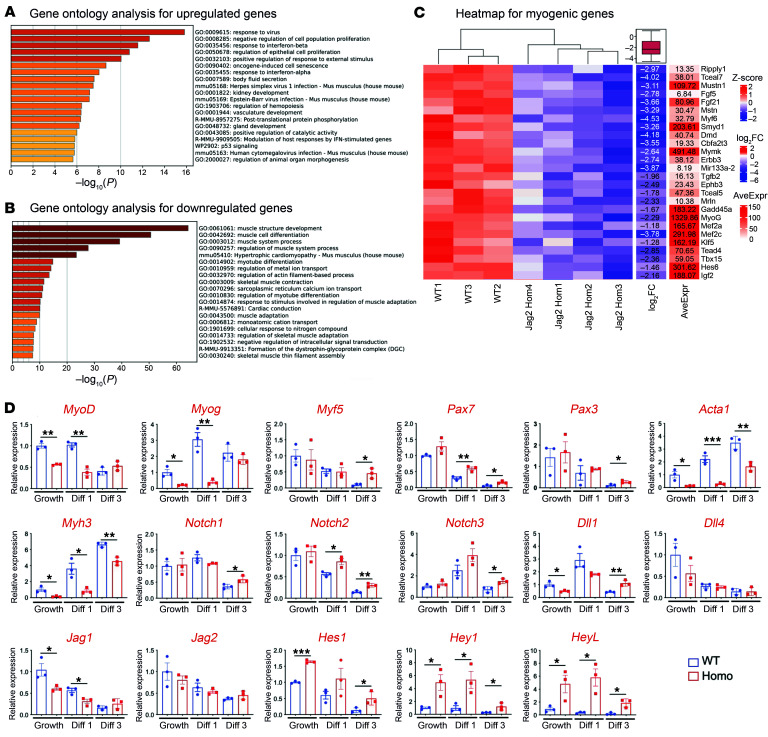
RNA-seq for gene expression profiles in *Jag2^sm^* versus WT MuSCs. (**A**) Metascape GO analysis reveals that numerous significantly upregulated genes in RNA samples from *Jag2^sm^* versus WT MuSCs are involved in negative regulation of cell proliferation. (**B**) GO analysis reveals that numerous significantly downregulated genes in RNA samples from *Jag2^sm^* versus WT MuSCs are muscle related. (**C**) Heatmap for downregulated genes associated with myogenic regulatory genes. (**D**) RT-qPCR was performed on WT and *Jag2^sm^* MuSCs under growth, day 1, and day 3 differentiation conditions to detect the expression of myogenic and NOTCH pathway–related genes. Unpaired 2-tailed Student’s *t* tests; *P < 0.05, **P < 0.01, and ***P < 0.001. Data are shown as the mean ± SEM; *n* = 3 biological replicates.

To probe global gene expression changes in *Jag2* deficiency, we performed whole-transcriptome sequencing (RNA-seq) on MuSCs isolated from the hind limb muscles of *Jag2^sm^* and WT mice. MuSCs were isolated using antibody-mediated magnetic sorting. Total RNA was isolated, reverse-transcribed to cDNA, and sequenced using a long-read sequencing platform from Oxford Nanopore Technologies. Metascape GO analysis revealed that 702 genes were significantly dysregulated (adjusted *P* value < 0.05) in homozygous *Jag2^sm^* compared with WT MuSCs. There were 186 upregulated genes and 516 downregulated genes (Figure 5, A and B, and Supplemental Tables 2 and 3). Metascape GO analysis indicated that 106 genes related to muscle structure development (GO:0061061), including 28 myogenic regulatory genes such as *Myog*, *Myf6*, *Mef2c*, *Mymk*, and *Igf2*, were downregulated in *Jag2^sm^* MuSCs (Figure 5, B and C, and Supplemental Tables 3 and 4). Negative regulators of cell proliferation, including *Cxcl12* and *Sox4*, were identified in the upregulated genes (Figure 5A and Supplemental Table 2). Among NOTCH receptor genes, *Notch1*, *Notch2*, and *Notch3* were expressed in WT MuSCs, and *Notch2* expression was upregulated in *Jag2^sm^* MuSCs (Supplemental Table 5). *Dll1*, *Jag1*, and *Jag2* were detected in both WT and *Jag2^sm^* MuSCs (Supplemental Table 5), suggesting dysregulation of NOTCH signaling in *Jag2^sm^* MuSCs. Reduced myogenic differentiation and expression of NOTCH receptor and ligand genes were confirmed in *Jag2^sm^* MuSCs via RT-qPCR (Figure 5D). These findings are consistent with observed defects in MuSC proliferation and myogenic differentiation in *Jag2* deficiency. Increased expression of the NOTCH downstream effector genes *Hes1*, *Hey1*, and *Heyl* was observed in *Jag2^sm^* compared with WT MuSCs (Figure 5D).

### Cis-inhibition of NOTCH signaling by JAG2 in MuSCs.

**Figure 6:**
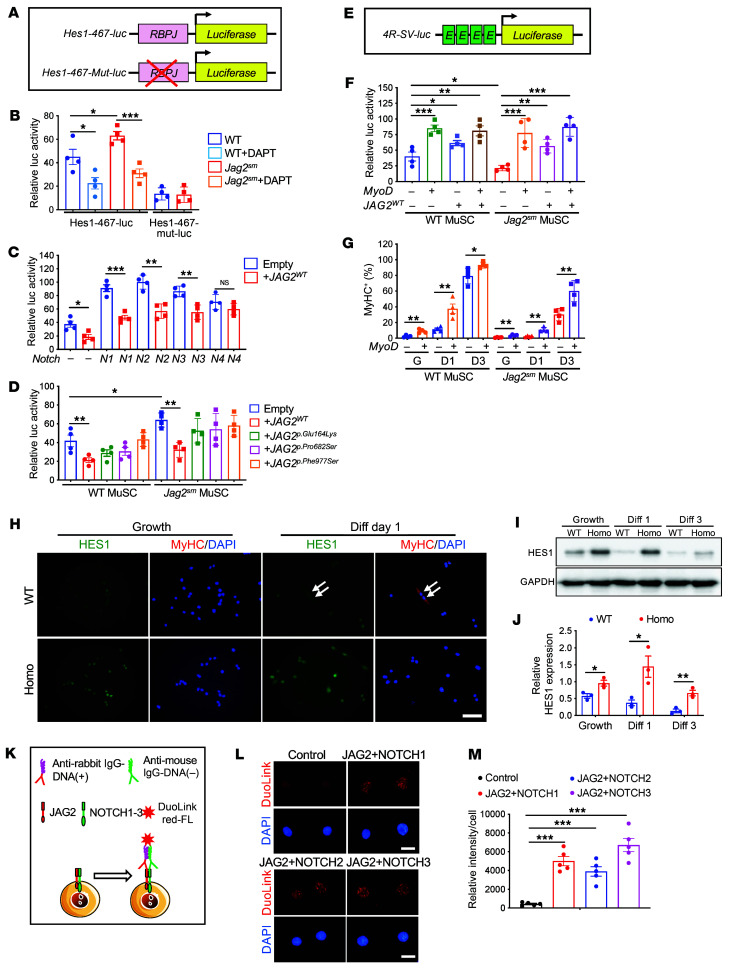
Human *JAG2* suppresses NOTCH signaling and promotes myogenesis. (**A**) *Luc* vectors (*Hes1-467-Luc* and *Hes1-467-Mut-Luc*) were used for NOTCH activities. (**B**) Homozygous *Jag2^sm^* MuSCs show higher *Hes1-467-Luc* activity versus WT MuSCs. *Luc* activities were diminished by mutation of the RBP-J binding site or by treatment with DAPT. (**C**) Expression of *Notch1-4* (*N1-4*) increased *Hes1-467-Luc* activities that were suppressed by human *JAG2* in WT and homozygous *Jag2^sm^* MuSCs. (**D**) Human reference *JAG2* but not pathogenic *JAG2* variants suppressed Luc activities. (**E**) *4R-SV-Luc* contains 4x E-boxes for consensus binding sites for MYOD. (**F**) Expression of *MyoD* and human *JAG2* activates *4R-SV-Luc* in MuSCs. (**G**)*MyoD* promoted MyHC^+^ myogenic differentiation in MuSCs in growth (G) and differentiation conditions (days 1 and 3). (**H**) HES1 expression is higher in homozygous *Jag2^sm^* versus WT MuSCs but downregulated in MyHC^+^ myocytes (arrows). (**I**) Western blotting for HES1 in MuSCs under growth and differentiation day 1 and 3 conditions. GAPDH was used as an internal control for loading. (**J**) Western blotting showed increased amounts of HES1 in homozygous *Jag2^sm^* MuSCs versus WT MuSCs. (**K**) The diagram shows a DuoLink PLA for a protein complex of JAG2 and NOTCH1, NOTCH2, or NOTCH3 within MuSCs. Anti-JAG2 and anti–NOTCH1–3 antibodies were used followed by secondary IgG with (+) and (–) strands of oligo DNAs. Red fluorescence tags were incorporated with successful ligation. (**L**) In DuoLink labeling, patchy red complexes were observed around the cell membrane but not in the control (no antibodies). (**M**) Quantification of DuoLink^+^ intensity was performed. DAPI stained all nuclei (blue). Scale bars: 100 μm (**H**), 10 μm (**L**). One-way ANOVA followed by Bonferroni’s post hoc tests and unpaired 2-tailed Student’s *t* tests; *P < 0.05, **P < 0.01, and ***P < 0.001. Data are shown as the mean ± SEM; *n* = 4 (**B**–**G**), *n* = 3 (**J**), and *n* = 5 (**M**) biological replicates.

To determine whether human JAG2 suppresses NOTCH signaling in MuSCs via *cis-*inhibition, we cotransfected *JAG2* with the NOTCH reporter gene *Hes1-467-Luc*, containing the 467 bp *Hes1* gene upstream region, or with *Hes1-467-Mut-Luc*, which lacks a RBP-J binding site essential for assembly of the transcriptional complex with NOTCH intracellular domains and other binding partners and subsequent NOTCH target gene activation (Figure 6A). *Hes1-467-Luc* activity was elevated in *Jag2^sm^* MuSCs, then abolished when the reporter gene with the mutant RBP-J binding site was used (Figure 6B). NOTCH reporter activation was blunted by *N*-[*N*-(3,5-difluorophenacetyl)-l-alanyl]-*S*-phenylglycine *t*-butyl ester (DAPT), a global γ-secretase/NOTCH inhibitor (40). These data indicate that *Jag2* deficiency promotes NOTCH signaling in MuSCs. To determine which NOTCH receptors are targeted by JAG2, the *Hes1-467-Luc* vector was cotransfected with human *JAG2* expression vector and *Notch* expression vectors. *JAG2* suppressed NOTCH1, NOTCH2, and NOTCH3 but not NOTCH4-mediated NOTCH reporter gene activation (Figure 6C). Given that NOTCH1, NOTCH2, and NOTCH3 are detected in quiescent and activated MuSCs, JAG2-mediated NOTCH signaling in MuSCs might be mediated through these NOTCH receptors. Since *JAG2* suppresses NOTCH signaling in MuSCs while *Jag2^sm^* MuSCs shows increased NOTCH signaling, JAG2 may suppress NOTCH signaling via *cis-*inhibition. WT and *Jag2^sm^* MuSCs transfected with reference *JAG2* but not variant *JAG2* (*p.Glu164Lys*, *p.Pro682Ser*, or *p.Phe977Ser*) showed reduced NOTCH signaling, confirming that *JAG2* pathogenic variants lack *cis-*inhibitory effects in MuSCs (Figure 6D and Supplemental Figure 4).

*Jag2^sm^* MuSCs display reduced myogenic differentiation but increased NOTCH activity and, consequently, reduced muscle regeneration after injury. To determine whether MYOD activity is downregulated in *Jag2^sm^* MuSCs, we tested the luciferase activity of the MYOD-binding site-driven *4R-SV-Luc* reporter gene. *4R-SV-Luc* incorporates 4x E-box elements and MYOD-binding motifs, which are sourced from the enhancer region of the muscle creatine kinase (*Ckm*) gene (Figure 6E) (41). MYOD activity is lower in *Jag2^sm^* MuSCs (Figure 6F). Cotransfection with MYOD promotes luciferase activity in *Jag2^sm^* MuSCs. *JAG2* promoted luciferase activities in both WT and *Jag2^sm^* MuSCs. *MyoD* overexpression rescued myogenic differentiation of *Jag2^sm^* MuSCs, as indicated by an increase in MyHC^+^ myocytes (Figure 6G and Supplemental Figure 5). Increased HES1 protein was detected in *Jag2^sm^* compared with WT MuSCs during growth and differentiation (Figure 6, H–J). The HES1 expression was abolished in MyHC^+^ myocytes in WT MuSCs (Figure 6H). These results strongly indicate that JAG2 is a myogenic promoter that acts as a *cis-*inhibitory factor for NOTCH signaling.

Using DuoLink technology, an in situ proximity ligation assay (PLA) that identifies 2 proteins in close proximity, we demonstrated the presence of JAG2-NOTCH1, -NOTCH2, and -NOTCH3 complexes on the MuSC plasma membrane (Figure 6, K and L). The quantity of DuoLink reaction products was measured, confirming the existence of JAG2-NOTCH1, -NOTCH2, and -NOTCH3 complexes exclusively when specific antibodies were used (Figure 6M). These findings suggest that JAG2 mediates *cis-*inhibition in MuSCs through direct interaction with NOTCH on the same cell.

### In vitro rescue effects of human reference JAG2 versus variant JAG2 in MuSCs.

**Figure 7:**
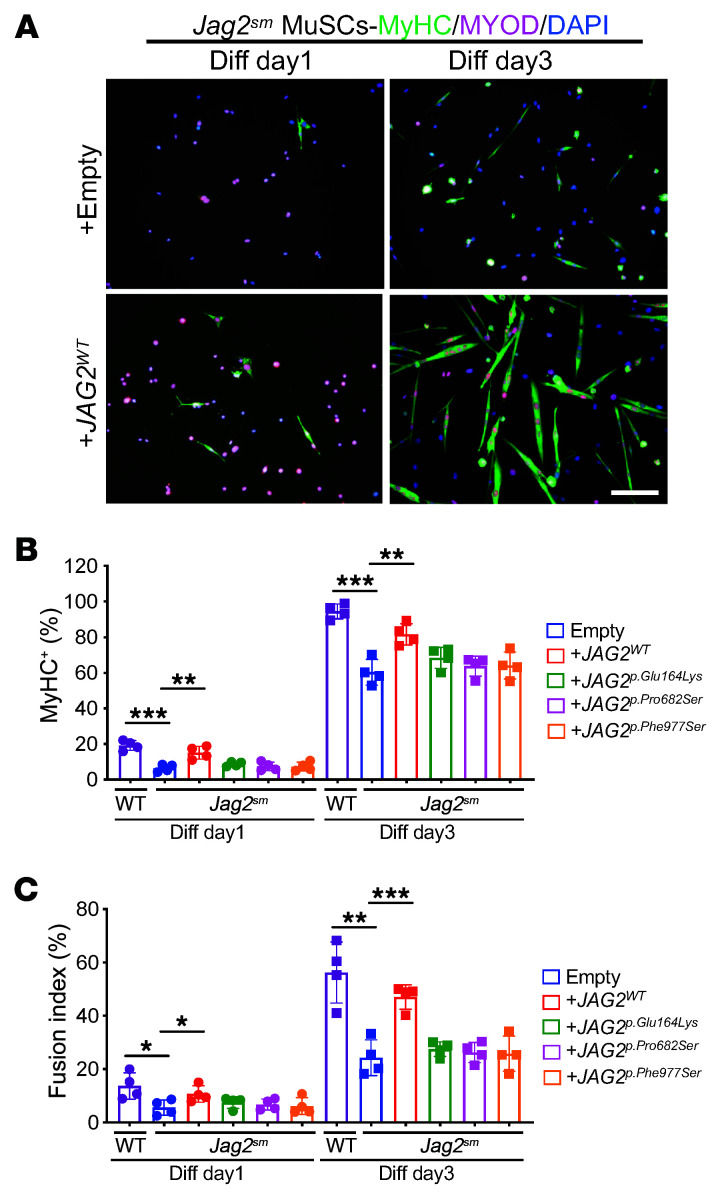
Overexpression of human *JAG2* rescues differentiation defects in *Jag2^sm^* MuSCs. (**A**) MuSCs isolated from homozygous *Jag2^sm^* mice were used for expression vector–mediated human *JAG2* overexpression (MyHC, green; MYOD, purple). (**B** and **C**) Overexpression of WT human *JAG2* (*JAG2^WT^*) but not human *JAG2* pathogenic variants (*p.Glu164Lys*, *p.Pro682Ser*, and *p.Phe977Ser*) increased MyHC^+^ myogenic differentiation (**B**) and fusion in day 1 and 3 differentiation conditions (**C**) versus control empty vector–transfected cells. DAPI stained all nuclei (blue). Scale bar: 100 μm. One-way ANOVA followed by Bonferroni’s post hoc tests; *P < 0.05, **P < 0.01, and ***P < 0.001. Data are shown as the mean ± SEM; *n* = 4 biological replicates.

Following culturing in differentiation media for 1 or 3 days, overexpression of human reference *JAG2* rescued myogenic differentiation defects in *Jag2^sm^* MuSCs, while overexpression of 3 human *JAG2* pathogenic variants (*p.Glu164Lys*, *p.Pro682Ser*, and *p.Phe977Ser*) (20) did not (Figure 7, A–C, and Supplemental Figure 5). Similarly, overexpression of reference *JAG2* but not variant *JAG2* (*p.Glu164Lys*, *p.Pro682Ser*, or *p.Phe977Ser*) rescued myogenic cell fusion in *Jag2*-deficient C2C12 myoblasts (Supplemental Figure 6A). These results demonstrate the essential roles of *JAG2* in MuSC function, providing evidence for a loss-of-function (LOF) pathogenic mechanism of *JAG2* variants.

### JAG2-mediated regulation of MuSC self-renewal via MuECs.

**Figure 8:**
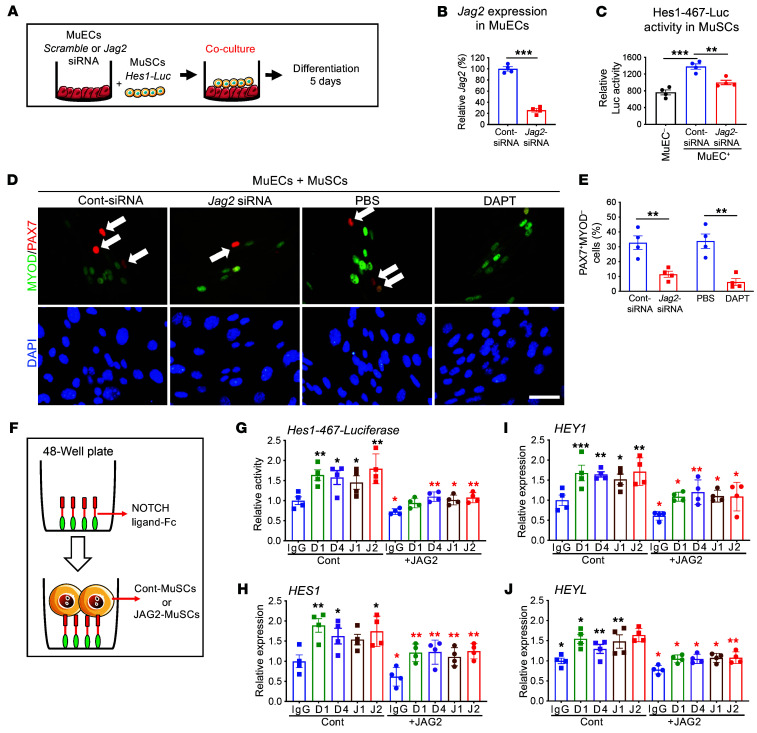
MuSC and MuEC coculture experiments. (**A**) MuSCs were transfected with *Hes1-467-Luc* (*Hes1-Luc*) and layered on top of the MuECs with scrambled or *Jag2* siRNA, allowed to adhere, and then cocultured in differentiation medium for 5 days. (**B**) MuECs transfected with *Jag2* siRNA show a significant reduction of *Jag2* mRNA expression versus scrambled siRNA. (**C**) Luc activities in MuSCs were increased when cocultured with MuECs versus MuSC alone or cocultured with MuECs with *Jag2* knockdown. (**D** and **E**) PAX7^+^MYOD^–^ self-renewing reserve cells were reduced when *Jag2* was cocultured with *Jag2*-KD MuECs versus control MuECs (arrows). Downregulation of NOTCH signaling through the pan-NOTCH inhibitor DAPT reduced the number of PAX7^+^MYOD^–^ self-renewing MuSCs versus PBS-treated cells in the cocultures (arrows). (**F**) Diagram of the evaluation of MuSCs treated with NOTCH ligands. (**G**) *Hes1-467-Luc* activity was assessed in control and *JAG2*-expressing MuSCs exposed to NOTCH ligand (control-IgG-Fc, DLL1-Fc, DLL4-Fc, JAG1-Fc, and JAG2-Fc). (**H**–**J**) Comparative mRNA expression levels of the NOTCH effector genes *Hes1* (**H**), *Hey1* (**I**), and *HeyL* (**J**) in control and *JAG2*-expressing MuSCs exposed to NOTCH ligand (control-IgG-Fc, DLL1-Fc, DLL4-Fc, JAG1-Fc, and JAG2-Fc). Each ligand-Fc was versus control IgG-Fc (black asterisks). Each ligand-Fc overexpressing *JAG2* was versus ligand-Fc lacking *JAG2* (red asterisks). DAPI stained all nuclei (blue). Scale bar: 50 μm. One-way ANOVA followed by Bonferroni’s post hoc tests and unpaired 2-tailed Student’s *t* tests; *P < 0.05; **P < 0.01, and ***P < 0.001. Data are shown as the mean ± SEM; *n* = 4 biological replicates.

MuECs play an essential role in MuSC self-renewal during muscle regeneration (6, 14), but the exact signaling mechanism between the 2 cell types is unclear. We examined JAG2-mediated effects of MuECs on MuSC self-renewal. We confirmed *Jag2* expression in MuECs and MuSCs via RNA-seq (Figure 1A), histology using a *Jag2^LoxP/LoxP^* mouse line with a *LacZ/Neo* cassette that enabled us to detect *Jag2*-expressing cells (Figure 1B), RT-qPCR (Figure 1C), and immunostaining (Figure 1D) in muscle and dissociated cells. We performed coculture experiments using MuECs and MuSCs isolated from WT mice. MuECs were transfected with either *Jag2* or scrambled control siRNA (Figure 8B), and their ability to support MuSC self-renewal was evaluated by quantifying PAX7^+^/MYOD^–^ reserve cells (i.e., self-renewing MuSCs or reserve cells) after 5 days of coculture (Figure 8A). MuSCs cocultured with control showed increased *Hes1-467-Luc* activity compared with MuSC culture alone (Figure 8C), indicating MuEC-mediated upregulation of NOTCH signaling in MuSCs. By contrast, *Jag2*-depleted MuECs exhibited reduced *Hes1-467-Luc* activity and PAX7^+^/MYOD^–^ self-renewing reserve cells compared with those cocultured with control MuECs (Figure 8, C–E). This reduced self-renewing effect was replicated in cocultures treated with DAPT (Figure 8, D and E), suggesting that JAG2-mediated NOTCH activation in MuSCs is required for self-renewal. These results demonstrate that JAG2 in MuECs regulates MuSC self-renewal through *trans-*activation, in addition to the cell-autonomous *cis-*inhibition effects of JAG2 (Table 1). *Jag2* depletion in MuECs also showed increased *Hes1-467-Luc* activity. When *JAG2* was overexpressed in MuECs, the *Hes1-467-Luc* activities were decreased, suggesting the similar JAG2-mediated cell-autonomous *cis*-inhibition of NOTCH in MuECs (Supplemental Figure 6B). However, secretome RT-qPCR analysis demonstrated no significant expression differences between control MuECs and *Jag2*-depleted MuSCs (Supplemental Figure 6C).

To determine whether extracellular JAG2 can *trans-*activate endogenous NOTCH activity and whether that activity is suppressed by intracellular JAG2 via *cis-*inhibition, WT MuSCs were plated onto dishes that were treated with NOTCH ligands linked to the Fc domain of IgG: DLL1-Fc, DLL4-Fc, JAG1-Fc, and JAG2-Fc (Figure 8F). Anti-goat IgG was used as a control. DLL-Fc, DLL4-Fc, JAG1-Fc, and JAG2-Fc increased *Hes1-467-Luc* activities, which were significantly suppressed by *JAG2* expression in WT MuSCs (Figure 8G), prompting elevated expression of the NOTCH effector genes *Hes1*, *Hey1*, and *HeyL* compared with control Fc treatment (Figure 8, H–J). These extracellular NOTCH ligands mediated the upregulation of *Hes1*, *Hey1*, and *HeyL*, which was significantly suppressed by *JAG2*. Finally, JAG2-Fc clearly increased the number of PAX7^+^MYOD^–^ reserve cells compared with anti-goat IgG–treated cells in MuSC cultures (Supplemental Figure 6, D and E). Therefore, JAG2 can *trans-*activate NOTCH signaling and promote self-renewal in MuSC cultures, which are suppressed by intracellular JAG2-mediated *cis-*inhibition.

### MuEC-derived Jag2 is essential for MuSC self-renewal, while MuSC-derived Jag2 is essential for proper MuSC myogenic differentiation.

**Figure 9:**
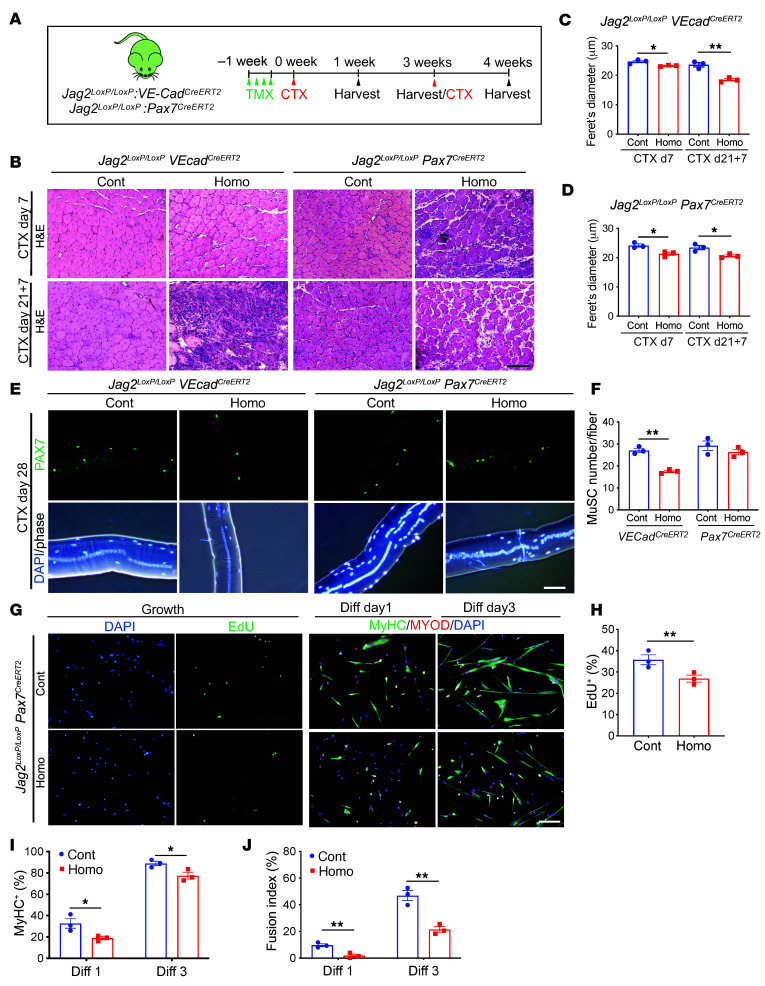
Reduced self-renewal in *Jag2^LoxP/LoxP^ VEcad^CreERT2^* and reduced regeneration in *Jag2^LoxP/LoxP^ Pax7^CreERT2^* mice. (**A**) Following TMX injection, single or repeated CTX injections into the TA muscle were performed. (**B**) H&E staining of TA by 7 days following CTX injection and 21+7 days following sequential CTX injections in *Jag2^LoxP/LoxP^ VEcad^CreERT2^* and *Jag2^LoxP/LoxP^ Pax7^CreERT2^* mice. Scale bar: 100 μm. (**C** and **D**) Feret’s diameters of TA muscle fibers in *Jag2^LoxP/LoxP^ VEcad^CreERT2^* and *Jag2^LoxP/LoxP^ Pax7^CreERT2^* mice following CTX injections. (**E** and **F**) Single-muscle fibers were isolated at 28 days following CTX injection. Anti-PAX7 antibody staining shows a reduced number of self-renewing MuSCs in *Jag2^Δ/Δ^ VEcad^CreERT2^* but not in *Jag2^Δ/Δ^ Pax7^CreERT2^* mice. (**G**–**J**) *Jag2^Δ/Δ^ Pax7^CreERT2^* MuSCs with TMX treatment (**G**) show reduced EdU^+^ proliferating cells (green) in growth (**H**), MyHC^+^ myogenic differentiation (**I**; green), and fusion index (**J**) in day 1 and 3 of differentiation conditions versus control cells. DAPI stained all nuclei (blue). Scale bars: 50 μm (**E**), 100 μm (**G**). Unpaired 2-tailed Student’s *t* tests; *P < 0.05 and **P < 0.01. Data are shown as the mean ± SEM; *n* = 3 biological replicates.

Our RNA-seq analysis and in vitro experiments demonstrated that JAG2 regulates MuSC proliferation, myogenic differentiation, and self-renewal via both cell-autonomous *cis-*inhibition and MuEC-MuSC interaction–mediated *trans-*activation. To confirm these findings in vivo, we investigated the effects of MuEC- and MuSC-specific *Jag2* deletions mediated by *VE-cadherin^CreERT2^* and *Pax7^CreERT2^*, using the *Jag2^LoxP/LoxP^ VEcad^CreERT2^* and *Jag2^LoxP/LoxP^ Pax7^CreERT2^* mice, respectively, following TMX injection (Figure 1B and Figure 9A). TA muscles were harvested 7, 21, and 21+7 days following sequential CTX injections. Using qPCR, we verified efficient conditional *Jag2* gene deletion (*Jag2^Δ/Δ^*) in MuECs and MuSCs following Cre activation in *VEcad^CreERT2^ Jag2^LoxP/LoxP^ Pax7^tdT^* and *Pax7^CreERT2^ Jag2^LoxP/LoxP^ Pax7^tdT^* mice, respectively, compared with control *Jag2^+/+^* MuECs and MuSCs (Supplemental Figure 7). TA cross sections in *VEcad*-mediated *Jag2*–conditional KO (cKO) mice (*MuEC-Jag2^Δ/Δ^*) displayed reduced muscle regeneration 7 and 21 days following CTX injection (Figure 9, B and C, and Supplemental Figure 8, A and B). At 28 days following sequential CTX injections in *MuEC-Jag2^Δ/Δ^* mice, muscle regeneration was significantly impaired (Figure 9, B and C), suggesting defects in MuSC self-renewal. TA cross sections in *Pax7*-mediated *Jag2*-cKO (*MuSC-Jag2^Δ/Δ^*) mice displayed reduced muscle regeneration by 7 and 21 days following single CTX injections (Figure 9, B and D, and Supplemental Figure 8, A and B). At 28 days following sequential CTX injections in *MuSC-Jag2^Δ/Δ^* mice, muscle regeneration was slightly impaired (Figure 9, B and D). The number of MuSCs was significantly reduced in the regenerating TA muscle sections at 3 weeks and in isolated single-muscle fibers from *MuEC-Jag2^Δ/Δ^* mice compared with those in *MuEC-Jag2^+/+^* mice at 4 weeks following CTX injection (Figure 9, E and F, and Supplemental Figure 8, A and C), confirming that self-renewal of MuSCs is regulated by MuEC-derived JAG2 during muscle regeneration. Therefore, reduced MuSC self-renewal in MuEC-specific *Jag2*-KO mice indicates that JAG2 is essential for MuSC self-renewal via MuEC-mediated *trans-*effects during muscle regeneration (Table 1). However, isolated single-muscle fibers and TA muscle sections from *MuSC-Jag2^Δ/Δ^* mice following muscle injury showed similar numbers of MuSCs compared with those in *MuSC-Jag2^+/+^* mice (Figure 9, E and F, and Supplemental Figure 8, A and C), indicating that self-renewal of MuSCs is not regulated by MuSC-derived *Jag2* during muscle regeneration. No differences were observed in the number of CD31^+^ capillaries between control and homozygous muscle in *MuEC-Jag2^Δ/Δ^* and *MuSC-Jag2^Δ/Δ^* mice (Supplemental Figure 8, A and D). MuSCs cultured from *Jag2* MuSC-specific KO mice showed reduced proliferation and differentiation (Figure 9, G–J). We conclude that JAG2 is essential for MuSC differentiation via cell-autonomous *cis-*inhibition of NOTCH signaling during muscle regeneration (Table 1).

### Human JAG2 rescues Ser deficiency in Drosophila, but pathogenic variants do not.

**Figure 10:**
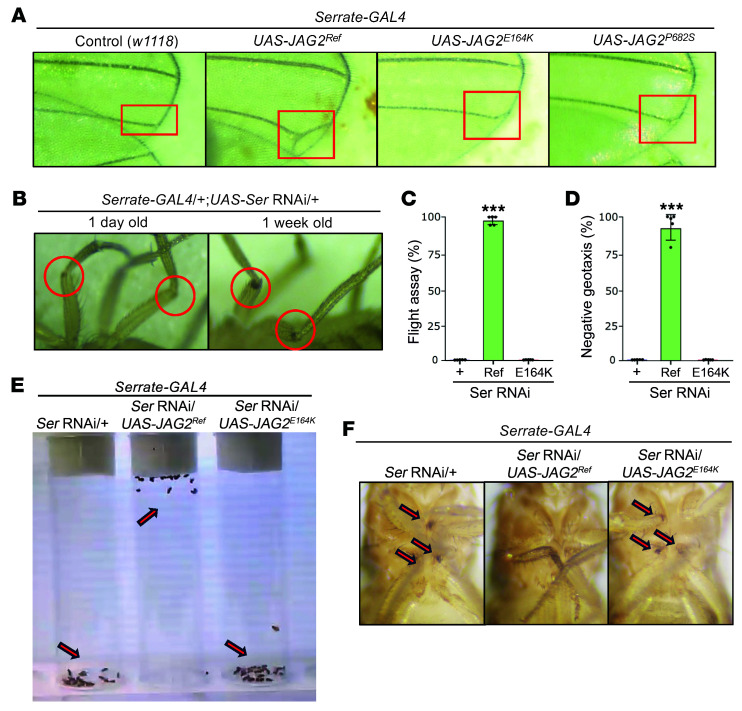
Reference human *JAG2* rescues *Ser* deficiency in *Drosophila*, while the human variant *JAG2^E164K^* does not. (**A**) Overexpression of reference human *JAG2* (*JAG2^Ref^*) in transgenic flies induced delta-shaped wing veins, but variants showed marginal effects. (**B**) RNAi-mediated *Ser* knockdown generated progressive melanotic spots on the legs (red circles). (**C**–**F**) Expression of *JAG2^Ref^* rescued manifestations of serrate deficiency, whereas expression of *JAG2^E164K^* did not, on measures of flight (**C**) and negative geotaxis (**D** and **E**), along with melanotic spots (**F**; red arrows). One-way ANOVA followed by Bonferroni’s post hoc tests; ***P < 0.001. Data are shown as the mean ± SEM; *n* = 5 biological replicates.

*Ser*, the *Drosophila* ortholog of human *JAG1* and *JAG2*, plays a critical role in wing development. In the wing disc, *Ser* is expressed in the epithelium adjacent to adult muscle precursors (AMPs), where it may promote the proliferation of muscle progenitors via *trans-*activation of NOTCH (42). *Ser* is also expressed in a distinct subset of AMPs (43). We generated transgenic fly lines carrying UAS-human *JAG2* constructs, allowing for tissue-specific expression of *JAG2* using the Gal4/UAS system. When reference human *JAG2* (*JAG2^Ref^*) was overexpressed using the *Ser-Gal4* driver mimicking the endogenous expression pattern of *Ser*, flies developed normally. However, the wings in the corresponding adult transgenic flies showed a characteristic “delta” wing vein phenotype (44), indicating a genetic interaction between human *JAG2* and the *Drosophila* *Delta*/*Notch* pathway (Figure 10A). Expression of either human pathogenic *JAG2* variant (*p.Glu164Lys* or *p.Pro682Ser*) associated with muscular dystrophy in humans (20) led to an attenuated wing vein delta phenotype, suggesting a LOF effect (Figure 10A). Likewise, our results in mice showed that the pathogenic *JAG2* variants result in LOF (Figures 2–4 and 9 and Table 1). RNAi suppressed endogenous *Ser* expression in *Ser*-expressing cells with *Ser-Gal4*. RNAi-mediated *Ser* knockdown did not induce developmental abnormalities, such as pupal lethality or eclosion defects. Right after eclosion, the *Ser* RNAi adult flies displayed normal walking and flight behavior. However, a rapid decline in locomotor activity occurred within a week after eclosion, including both flight and gait impairments (Figure 10, B–E). Flies with reduced *Ser* exhibited progressive development of dark melanotic spots, indicating tissue necrosis and a hemocyte-mediated inflammatory response. This is consistent with the need for NOTCH signaling in the leg imaginal discs to promote leg segment formation (45). The degenerative phenotypes observed with *Ser* LOF were useful to assess whether human *JAG2* could rescue the defects. Expression of reference human *JAG2*, but not *JAG2* *p.Glu164Lys*, in *Ser*-deficient flies rescued the locomotor deficits and necrotic legs (Figure 10, C–F). These findings demonstrate the functional conservation of *JAG2* across species and provide in vivo evidence for the LOF mechanism of pathogenic *JAG2* variants (Table 1).

## Discussion

Biallelic pathogenic variants in the canonical NOTCH ligand *JAG2* cause a form of muscular dystrophy (20, 46, 47). Pathogenic variants in the paralogous gene *JAG1* are associated with Alagille syndrome, which does not prominently involve skeletal muscles, yet *JAG1* augmentation shows promise as a therapeutic target for muscular dystrophy (48, 49). NOTCH signaling affects several biological functions associated with skeletal muscle, including MuSC self-renewal, maintenance, and muscle regeneration. JAG2 is expressed in both MuSCs and MuECs, but the significance of JAG2 for skeletal muscle development and health was not clear.

**Figure 11:**
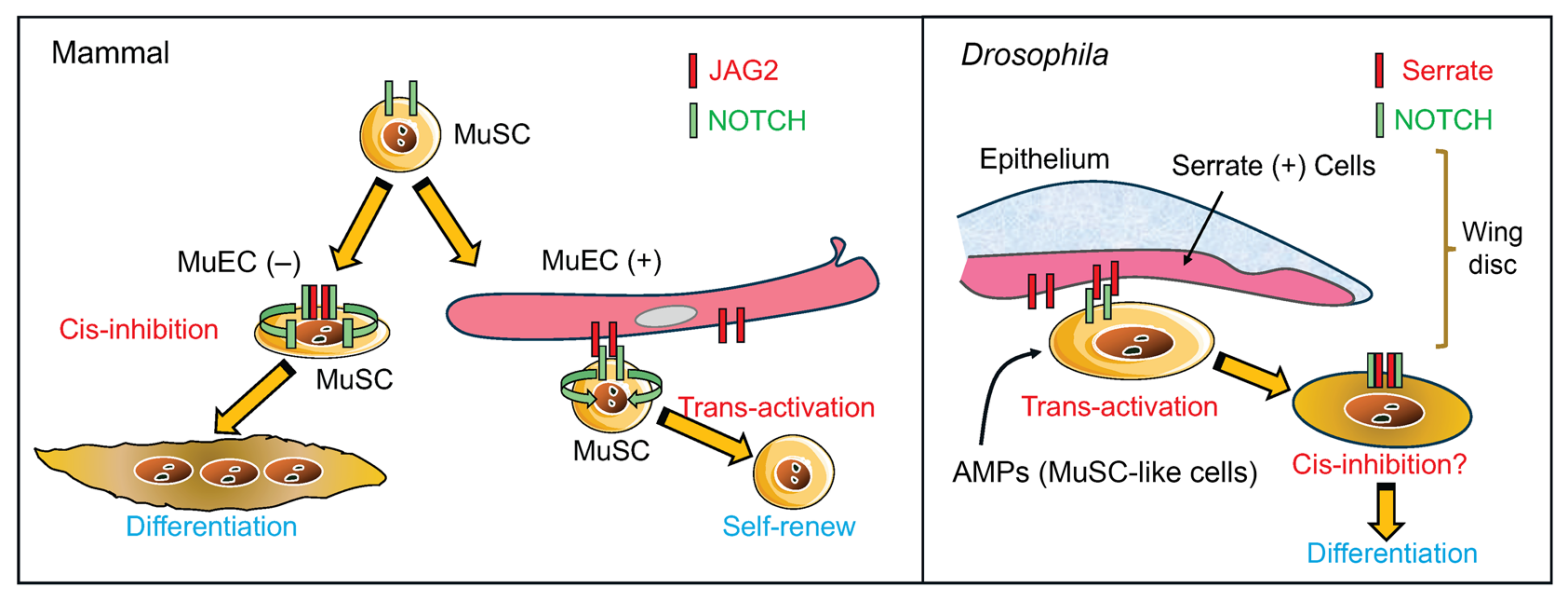
A diagram illustrating the role of *Jag2* expression in mammal and *Drosophila* muscles. In mammals (left), neighboring capillary MuECs *trans*-activate NOTCH signaling in MuSCs via *Jag2* for MuSC self-renewal. MuSCs, which do not receive *Jag2*-mediated *trans*-activation by MuECs, suppress NOTCH signaling via *cis-*inhibition by cell-autonomous *Jag2* expression, stimulating myogenic differentiation. In *Drosophila* wing discs (right), the ortholog *Ser* is expressed in epithelial cells, which activates NOTCH signaling in adjacent AMPs, which are MuSC-like cells, to maintain the progenitor pool. AMPs express *Ser*, but it is unclear whether *cis-*inhibition by *Ser* occurs in AMPs.

Our data indicate that *Jag2* deficiency in MuSCs impairs their myogenic differentiation potential via failed *cis-*inhibition effects for NOTCH activity, while *Jag2* deficiency in MuECs impairs MuSC self-renewal via failed *trans-*activation effects for NOTCH activity as niche cells, suggesting that JAG2-related cell-autonomous (*cis*) and cell-nonautonomous (*trans*) NOTCH signaling affects skeletal muscle development, regeneration, and health in different ways (Figure 11). In *Drosophila*, *Ser*-expressing niche cells in the wing epithelium regulate the proliferation and maintenance of AMPs (42). We demonstrated that in vivo knockdown of *Ser* (the fly ortholog of *JAG2*) in *Ser*-expressing cells resulted in motor function and morphological defects. These phenotypes are postulated to result from reduced *trans-*activation. It is unclear if *cis-*inhibitory activity is also involved in *Drosophila* adult muscle development (Figure 11).

Homozygous *Jag2* hypomorphic (*Jag2^sm^*) mice display digit and craniofacial developmental defects (50, 51). We showed that homozygous *Jag2^sm^* mice display impaired muscle regeneration due to a reduced PAX7^+^ MuSC population during muscle development. The surviving homozygous *Jag2^sm^* MuSCs showed reduced proliferation and decreased myogenic differentiation. To reveal whether MuSC defects seen in *Jag2^sm^* mice are due to the *trans-*effects via neighboring niche cells or cell-autonomous *cis-*effects, we utilized MuEC-MuSC coculture experiments, NOTCH ligand-Fc treatment cultures, and Cre recombinase–mediated *Jag2*-cKO mice. We demonstrated that MuEC-specific *Jag2* KO resulted in reduced MuSC self-renewal. Our MuEC and MuSC coculture experiments demonstrated that MuEC-derived JAG2 is required for sufficient MuSC self-renewal, underscoring the significance of direct cellular interaction between MuECs and MuSCs for the activation of NOTCH signaling in vitro and in vivo.

### Trans-activation of NOTCH signaling by JAG2.

Multiple investigations have implicated MuECs in the functionality of MuSCs (4–6, 52). We have demonstrated that vascular network enhancement augments MuSC populations in mouse models of Duchenne muscular dystrophy (7–9). Recent works, including our studies, revealed a molecular mechanism that connects MuECs and MuSCs, along with the functional outcomes of this signaling. We demonstrated that the proximity of MuSCs to capillaries is actively orchestrated by VEGFA secreted by MuSCs, attracting capillaries to create a juxtavascular environment for MuSCs (6). In addition, MuSC self-renewal is induced by NOTCH activation, which is stimulated by the adjacent capillary MuECs through as a *trans-*activator (14). By contrast, the MuEC-derived secreted form of DLL4 regulates muscle fiber atrophy (35). Our in vitro coculture experiments and in vivo conditional gene KO mice showed that MuEC-derived JAG2 is essential for MuSC self-renewal. Therefore, MuEC-mediated MuSC self-renewal requires at least 2 NOTCH ligands, DLL4 and JAG2.

Recent findings have underscored the necessity of NOTCH receptors for MuSCs to revert to quiescence and establish stem cell populations (53, 54). Regarding the neighboring cell origin for NOTCH activation, DLL4, derived from mature muscle fibers, activates NOTCH3 expression in MuSCs, facilitating their return to quiescence (54). Mature muscle fiber–derived DLL4 is important for the maintenance of quiescent MuSCs on muscle fibers in muscle fiber–specific *Dll4*-KO mice (36). Moreover, DLL4 from muscle fibers regulates NOTCH signaling in the proximal MuSCs to enhance their regenerative potential via increased self-renewal (55). We found that DLL4 and JAG2 levels were reduced in muscle fibers compared with MuECs, suggesting that MuECs play a crucial role as NOTCH ligand–synthesizing cells that support MuSC self-renewal in skeletal muscle. However, it is also possible that, similar to muscle fiber–derived DLL4, muscle fiber–derived JAG2 may also play a role in MuSC self-renewal and/or maintenance via activation of NOTCH signaling in MuSCs (36, 55).

### Ser-Notch–mediated Drosophila myogenic progenitor maintenance.

Using *Ser-GAL4* to knock down *Ser* in *Ser*-expressing cells, we observed previously unreported adult *Drosophila* phenotypes, which we believe are due to reduced *trans-*activation. Whether *Drosophila* *Ser* is also involved in *cis-*inhibition is unknown. Recent single-nucleus sequencing (56) suggests that a discrete population of *Ser*-expressing cells is present in the adult muscle system, although there are fewer of them than *Delta*-expressing cells and they do not coexpress *Delta*; thus, *Ser* remains the main *cis-*inhibitory signal in fruit flies.

### Cis-inhibition.

We demonstrated that MuSC-specific *Jag2* KO resulted in reduced myogenic differentiation without affecting MuSC self-renewal capacity. These results are consistent with our RNA-seq and gene knockdown data. NOTCH signaling relies on families of ligands and receptors that relay messages to adjacent cells in various combinations across distinct cell types, as seen in MuEC-MuSC interactions (in *trans*), and within the same MuSCs (in *cis*). NOTCH ligands and their corresponding receptors that are present within the same cell display *cis-*inhibition of NOTCH signaling (57). This *cis-*inhibition plays a crucial role in various developmental processes, such as wing disc formation in *Drosophila*, maintenance of epidermal stem cells, neurogenesis, pancreatic cell differentiation, and hematopoiesis (33, 34, 57, 58). A systematic examination of *cis-* and *trans-*interactions between JAG2 and different NOTCH receptors could yield more profound insights into cell–cell communication–mediated MuSC functions.

### NOTCH for muscular dystrophies.

Reduced MuSC self-renewal, maintenance, and differentiation contribute to the disease mechanism of muscular dystrophies. Therapies targeting NOTCH signaling could selectively enhance MuSC replication, potentially alleviating the symptoms of muscular dystrophy. Two other inherited muscle disease genes aside from *JAG2* have been linked to the NOTCH signaling pathway: *MEGF10* (21, 22) and *POGLUT1* (23). We determined that MEGF10 and NOTCH1 interact at their intracellular domains and that this interaction is impaired by pathogenic variants (59). Loss of NOTCH signaling in *POGLUT1* deficiency has been demonstrated in skeletal muscle tissue from affected individuals and in *Drosophila* (23). Preclinical data in dog, zebrafish, and mouse models of Duchenne muscular dystrophy show therapeutic potential for JAG1-mediated modulation of NOTCH signaling (48, 49). These findings, in tandem with the results of this study, indicate that JAG2 and the NOTCH signaling pathway in general are promising therapeutic targets for muscle disease. A small molecule screen could identify drug candidates for JAG2 augmentation, and a gene therapy approach could be developed for *JAG2*-related muscular dystrophy and other muscular dystrophies.

### Conclusions.

We have demonstrated that JAG2-mediated *trans-*activation and *cis-*inhibition of NOTCH signaling regulate MuSC function during muscle regeneration. JAG2 shows promise as a therapeutic target for muscular dystrophy, and our findings will help fine-tune interventions to focus on specific desirable downstream effects of JAG2-related interventions.

## Methods

### Sex as a biological variable.

Our study examined male and female animals, and similar findings are reported for both sexes.

### Mice.

C57BL/6N-*A^tm1Brd^* *Jag2^tm1a(KOMP)Wtsi^*/HMmucd (*Jag2^LoxP/LoxP^*; MMRRC:048257-UCD) mice were obtained from the Mutant Mouse Resource & Research Centers. *B6.Cg-Pax7^tm1(cre/ERT2)Gaka/J^* (*Pax7^+/CreERT2^*; JAX 017763) (39), *B6.Cg-Gt(ROSA)^26Sortm9(CAG-tdTomato)Hze/J^* (*Ai9*; JAX 007909) (60), *STOCK Jag2^sm^/J* (*Jag2^sm^*; JAX 000239) (37), and B6.129S4-Gt(ROSA)26Sor^tm2(FLP)*Sor^/J (*FLP*; JAX 012930) (61) mice were obtained from The Jackson Laboratory. *Kdr^tm2.1Jrt/J^* (*Flk1^+/GFP^*) mice were obtained from Masatsugu Ema (62). *Cdh5^+/CreERT2^* mice were obtained from Yoshiaki Kubota (63). *B6.Cg-Pax7^tm1(cre/ERT2)Gaka/J^* (*Pax7^+/CreERT2^*) mice were crossed with *B6.Cg-Gt(ROSA)^26Sortm9(CAG-tdTomato)Hze/J^* (*Ai9*) to yield the *Pax7^+/CreERT2^ R26R^tdT^*(*Pax7^tdT^*) mice (6, 39). *Pax7^tdT^* mice were bred with *Jag2^LoxP/LoxP^* and *Flk1^+/GFP^* to yield *Jag2^LoxP/LoxP^ Pax7^+/tdT^ Flk1^+/GFP^* mice. *Cdh5^+/CreERT2^* mice were crossed with *B6.Cg-Gt(ROSA)^26Sortm9(CAG-tdTomato)Hze/J^* (*Ai9*) to yield *Cdh5^+/CreERT2^ R26R^tdT^*(*Cdh5^tdT^*) mice. *Cdh5^tdT^* mice were bred with *Jag2^LoxP/LoxP^* and *Flk1^+/GFP^* to yield *Jag2^LoxP/LoxP^ Cdh5^+/tdT^* *Flk1^+/GFP^* mice. *Jag2^sm^* mice (51) were crossed with *Pax7^tdT^* to yield the *Jag2^sm^ Pax7^tdT^* mice. All mouse colonies were established (Table 1) and genotyped (Supplemental Table 6 and Supplemental Figure 7) in the laboratory. Cre recombination was induced using TMX (T5648, MilliporeSigma), 75 mg/kg body weight × 3 over 1 week at 3–6 weeks of age. *CreERT2* mice were used as controls. TA muscle regeneration was induced by intramuscular injection of 20 μL of 10 μM CTX (V9125, MilliporeSigma). The animals were housed in an SPF environment and monitored by Research Animal Resources of the University of Minnesota. All protocols (2204–39969A) were approved by the IACUC of the University of Minnesota and complied with NIH guidelines for the use of animals in research.

### Cell culture and immunostaining.

C2C12 myoblasts (CRL-1772) were obtained from American Type Culture Collection and cultured in DMEM medium with 10% FBS, 100 units/mL of penicillin, and 100 μg of streptomycin at 37°C in 5% O_2_ and 5% CO_2_. C2C12 cells were specific growth rate profiled to confirm their identity and tested negative for mycoplasma. MuSCs were isolated from adult mice (64, 65). After collagenase type II (CLS-2, Worthington) treatment, dissociated cells from mouse hind limb muscles were incubated with anti–CD31-PE (12-0311-82, eBiosciences), anti–CD45-PE (12-0451-81, eBiosciences), anti-Sca1-PE (A18486, eBiosciences), and anti–Integrin α7 (ABIN487462, MBL International), followed by anti-PE microbeads (130-048-801, Miltenyi Biotec), then underwent LD column (130-042-901, Miltenyi Biotec) separation. Negative cell populations were incubated with anti-mouse IgG beads (130-048-402, Miltenyi Biotec), and MS column (130-042-201, Miltenyi Biotec) separation was performed to isolate Integrin α7^+^ MuSCs. MuSCs were maintained in culture on collagen-coated plates in myoblast growth medium containing 20% FBS, 20 ng/mL bFGF (PHG0263, Invitrogen), 100 units/mL of penicillin, and 100 μg of streptomycin in Ham’s F10 medium. MuECs were isolated from adult mice (15, 66). Dissociated muscle cells were obtained as described above. Dissociated cells were incubated with CD45 MicroBeads (130-052-301, eBiosciences) and anti–CD45-PE (12-0451-81, eBiosciences) and underwent LD column (130-042-901, Miltenyi Biotec) separation. Negative cell populations were incubated with CD31 MicroBeads (130-097-418, Miltenyi Biotec), and MS column (130-042-201, Miltenyi Biotec) separation was performed to isolate CD45^–^CD31^+^ MuECs. MuECs were maintained in culture on fibronectin-coated plates in an EGM-2 Endothelial Cell Growth Medium-2 Bullet Kit (CC-3162, Lonza). All antibody-related materials are listed in Supplemental Table 7. All cell cultures were maintained in a humidified incubator at 37°C with 5% CO_2_ and 5% O_2_.

4-Hydroxy tamoxifen (H6278, MilliporeSigma) treatment (1 μM in EtOH) was used to induce *Jag2* deletion in MuSCs isolated from *Jag2^LoxP/LoxP^ Pax7^CreERT2^* mice. For the cell proliferation assay, cells were exposed to 1 μM EdU for 3 hours before being fixed and stained by the Click-iT EdU Alexa Fluor 488 or 647 Imaging Kit (C10337 or C10340, Thermo Fisher Scientific). To induce differentiation of MuSCs, the myoblast growth medium was replaced with differentiation medium that contained DMEM supplemented with 5% horse serum for 1–5 days. After cell cultures, anti-MyHC (MF20, DSHB) and anti-MYOD (C-20, Santa Cruz Biotechnology) antibodies followed by secondary anti–mouse-Alexa Fluor 488 (A-11001, Thermo Fisher Scientific), anti-rabbit–Alexa Fluor 647 (A-32795, Thermo Fisher Scientific), anti-MyHC (MF20), and anti-HES1 (D6P2U, Cell Signaling Technology) antibodies followed by secondary anti-mouse–Alexa Fluor 568 (A-11004, Thermo Fisher Scientific) and anti-rabbit–Alexa Fluor 488 (A-11008, Thermo Fisher Scientific) or anti-JAG2 antibody (C23D2, Cell Signaling Technology) followed by secondary anti-rabbit–Alexa Fluor 488 (A-11008) were used (Supplemental Table 7). LacZ expression in MuSCs obtained from *LacZ/Neo-Jag2^LoxP/LoxP^* mice was detected by X-gal staining overnight as described previously (67). Following X-gal staining, anti-PAX7 (DSHB) or anti-MYOD (C-20, Santa Cruz Biotechnology) antibodies followed by secondary anti-mouse–Alexa Fluor 488 (A-11001) or anti-rabbit–Alexa Fluor 488 (A-11008) were used. The fusion index (containing 2 or more nuclei in MyHC^+^ myotubes) was measured. For induction of apoptotic cell death in MuSCs, thapsigargin-mediated apoptosis was induced by 1 μM thapsigargin (T9033, MilliporeSigma) dissolved in EtOH for 24 hours. UV light–mediated apoptosis was induced by exposing the cells to UV light in a cell culture hood for 1 minute without medium. After UV exposure, cell survival was assessed 24 hours following culture in 0.1% FBS in Ham’s F10 medium using the Annexin V Assay Kit (ab232855, Abcam) and TUNEL staining kit (G7360, Promega). DAPI (0.1 μg/mL in PBS) was used for nuclei staining.

### Knockdown of Jag2 in C2C12 mouse myoblasts, MuSCs, and MuECs.

C2C12 cells were transfected with a cocktail of shRNA plasmids (Genecopoeia) against mouse *Jag2* using Lipofectamine 3000 (L3000001, Thermo Fisher Scientific). The *Jag2* and scrambled shRNA sequences are shown in Supplemental Table 6. shRNA transfection and positive clone selection were performed as described (20). *Jag2* siRNAs (sc-39673, Santa Cruz Biotechnology) and control scramble siRNA-A (sc-37007, Santa Cruz Biotechnology) were transfected in WT MuSCs or MuECs using Polyjet (11668019, Thermo Fisher Scientific), and cells were harvested on growth and differentiation day 1. *Jag2* downregulation was confirmed by RT-qPCR. Anti-MyHC (DSHB) antibody followed by secondary anti-mouse–Alexa Fluor 488 (A-11029, Thermo Fisher Scientific) antibody was used to detect differentiating myocytes. *Jag2*-knockdown MuECs were used for luciferase assays after transfection with *Hes1-467-Luc* and *JAG2* expression vectors. Endothelial secretome analysis was performed by RT-qPCR in *Jag2*-knockdown MuECs. DAPI was used for nuclear staining.

### NOTCH plate array and myotube-fusion index of differentiated C2C12 cells.

Scrambled and *Jag2* shRNA cells were switched at 80% confluence from normal growth medium containing 10% FBS to differentiation medium containing 2% horse serum and differentiated for 7 days. Total RNA was isolated using an RNA isolation kit (Zymo Research). Reverse transcription of mRNA was performed using a high-capacity RNA to cDNA kit (Applied Biosystems). qPCR-based gene expression analysis was conducted using the TaqMan Fast Advanced Master Mix in the QuantStudio 3 Real-Time PCR System (Thermo Fisher Scientific). The Taqman probes used were as follows: mouse *Jag2* (Mm01325629_m1), mouse *MyoG* (Mm00446194_m1), human *JAG2* (Hs99999198_m1), and mouse *Gapdh* (Mm99999915_g1) from Thermo Fisher Scientific. The cDNA samples were also examined via the TaqMan Array Mouse NOTCH Signaling Pathway, Fast 96-well plate (Thermo Fisher Scientific) containing a set of NOTCH signaling pathway–associated genes and endogenous control genes as reported previously (20). For myotube fusion index analysis, the cells were fixed in 4% paraformaldehyde (PFA) for 15 minutes and blocked in serum medium for 1 hour. The cells were stained with MyHC (MF20) primary antibody for 1 hour. Cells were then stained with anti-rabbit– or anti-mouse–Alexa Fluor 568 secondary antibody for 1 hour. Nuclei were stained using DAPI (D1306, Thermo Fisher Scientific), and the coverslips were mounted using Fluoromount Aqueous Mounting Medium (MilliporeSigma). The slides were imaged using a DM6000B or DM5500B epifluorescence microscope (Leica). The myotube-fusion index was determined by counting the number of nuclei within MyHC^+^ myotubes divided by the total number of nuclei in the field of view using ImageJ (NIH).

### Site-directed mutagenesis and overexpression of JAG2 variants in C2C12 myoblasts and MuSCs.

Site-directed mutagenesis was performed on the *JAG2* coding sequence in the *pCDNA3.1* backbone to generate the variants of interest using the Q5 Site-Directed Mutagenesis Kit (New England Biolabs). *pCDNA3.1JAG2* was a gift from Sandra Coppens. Mutagenic primers harboring the desired variants were used in a PCR reaction with WT *JAG2* sequence as the template. PCR was performed using the primers for the indicated *JAG2* variants (*p.Glu164Lys*, *p.Pro682Ser*, and *p.Phe977Ser*) (Supplemental Table 6). Generation and stable overexpression of the *JAG2* human variants in scrambled and *Jag2* shRNA cells were performed via selection with G418 (200 μg/mL).

### RNA and genomic DNA isolation and qPCR.

Cultured cells were washed with ice-cold PBS and lysed in place with TRIzol (15596018, Thermo Fisher Scientific). RNA was isolated using the DirectZol RNA Microprep Kit (R2062, Zymo Research) with on-column DNase digestion followed by cDNA synthesis using the Transcriptor First Strand cDNA synthesis kit (04379012001, Roche Molecular Diagnostics) with random primers. Genomic DNA for genotyping was isolated from mouse tail snips with lysis buffer containing Proteinase K (P2308, MilliporeSigma). qPCR was performed using GoTaq qPCR Master Mix (A6002, Promega). The input RNA amount was normalized across all samples, and *18S rRNA* was used for normalization of qPCR across samples. All primers were synthesized as custom DNA oligos from Integrated DNA Technologies (Supplemental Table 6).

### Single-muscle fiber isolation and staining.

Extensor digitorum longus muscle was dissected from uninjured or CTX-injected muscle and digested with 0.2% collagenase type-I (C0130, MilliporeSigma) for single-muscle fiber isolation (7). Single-muscle fibers were fixed with 2% PFA/PBS, permeabilized with 0.2% Triton X-100. Anti-PAX7 antibody (DSHB), followed by secondary anti-mouse–Alexa Fluor 488 (A-11029), was used for immunostaining (Supplemental Table 7). At least 20 single-muscle fibers per each mouse were used for the measurement. DAPI was used for nuclear staining.

### Luciferase reporter assays.

*Hes1-467-Luc* and *Hes1-467-Mut-Luc* were obtained from Addgene (catalog 41723 and 43805). *4R-SV-Luc* was obtained from Andrew Lassar (41). *pRL-TK* (E1910, Promega) was used as an internal control. *Hes1-467-Luc* was transfected to control MuECs and *Jag2*-depleted MuSCs. WT and homozygous MuSCs were transfected with expression vectors for human *JAG2* (*pcDNA3-JAG2*), mouse *MyoD* (*pcDNA3-MyoD*), mouse *Notch1* (*pCS2-Notch1*), mouse *Notch2* (*pPB[Exp]-Notch2*, VectorBuilder), human *NOTCH3* (*pPB[Exp]-NOTCH3*, VectorBuilder), mouse *Notch4* (*pHyTc-Notch4*, Addgene), or empty vectors and the luciferase reporter genes using PolyJet In Vitro DNA Transfection Reagent (SL100688, SignaGen Laboratories). Cells were harvested 48 hours after transfection. Luciferase activity was measured with a plate reader (LD400, Beckman Coulter) using a dual-luciferase reporter assay system (E1910, Promega).

### Ligand-coating NOTCH signaling assay.

A total of 5 × 10^4^ WT MuSCs, *Jag2^sm^* homozygous MuSCs, WT MuSCs carrying *JAG2*, and *Jag2^sm^* homozygous MuSCs carrying *JAG2* were placed in a 48-well tissue culture plate pretreated with 5 μg/mL of DLL1-Fc, DLL4-Fc, JAG 1-Fc, or JAG2-Fc (5026-DL, 10089-D4, 10969-JG, and 4748-JG, R&D Systems) and allowed to settle at room temperature for 1 hour. After 16–18 hours, the cells were transfected with *Hes1-467-Luc* using PolyJet In Vitro DNA Transfection Reagent (SL100688). After 3 hours, the medium was changed to growth medium for an additional 48 hours or differentiation medium for an additional 120 hours to detect PAX7^+^MYOD^–^ reserve cell population. For immunostaining, anti-PAX7 (DSHB) and anti-MYOD (C-20, Santa Cruz Biotechnology) antibodies followed by secondary anti-mouse–Alexa Fluor 488 (A-11001) or anti-rabbit–Alexa Fluor 488 (A-11008) were used.

### Histology and immunostaining for sections and cell cultures.

The mouse TA muscle was used for all histological analyses. Tissues were snap-frozen using LiN_2_ chilled isopentane and stored at –80°C. For histological analysis, 8μm thick transverse cryosections were used. Cryosections (8 μm thick) were used for H&E (7), Sirius red (Direct Red 80, 365548, MilliporeSigma) for fibrosis (8), and Oil Red O staining (O1391-250ML, MilliporeSigma) (64). LacZ expression in whole muscle and MuSCs obtained from *LacZ/Neo-Jag2^LoxP/LoxP^* mice was detected by X-gal staining overnight as described previously (7, 66). Muscle sections obtained from X-gal–stained muscle were used for anti-CD31 antibody staining followed by anti-rat–Alexa Fluor 488 (A-11006, Thermo Fisher Scientific). Anti-eMyHC (F1.652, DSHB) or anti-PAX7 (DSHB) and anti-Laminin (L0663, MilliporeSigma) antibodies, followed by anti-mouse–Alexa Fluor 488 (A-11001) and anti-rat–Alexa Fluor 568 (A-11077, Thermo Fisher Scientific) antibodies were used to detect MuSCs or regenerating muscle fibers, respectively. For capillary density measurement, anti-CD31 antibody (550274, BD Biosciences) and anti-Laminin antibody (L9393, MilliporeSigma) were used for TA sections, followed by anti-rat–Alexa Fluor 488 (A-11006) and anti-rabbit–Alexa Fluor 568 (A-11011, Thermo Fisher Scientific). Immunostaining was performed on 35 mm tissue culture plates or 8-well Permanox Chamber slides (C7182-1PAK, MilliporeSigma). Cells were fixed with 2% PFA for 5 minutes and immunostained (15). Cells were permeabilized with 0.2% Triton X-100 in PBS, blocked with 1% BSA in PBS, and incubated with primary antibodies followed by secondary antibodies. PBS with 0.01% Triton X-100 was used for washing cells. Nuclei were counterstained with DAPI. The antibodies are listed in Supplemental Table 7. Microscopy images were captured with a DP-1 digital camera attached to a BX51 fluorescence microscope with ×10, ×20, or ×40 UPlanFLN objectives with cellSens Entry 1.11 (Olympus). Photoshop (Adobe) and Fiji (NIH) were used for image processing and enumerating Feret’s diameters (7).

### Overexpression experiments.

The *pcDNA3* expression vectors for WT human *JAG2* (*pcDNA3-JAG2*), human *JAG2* pathogenic variants (*p.Glu164Lys*, *p.Pro682Ser*, and *p.Phe977Ser*), and mouse *MyoD* were transfected to low-passaged (2–3 passages) WT or homozygous *Jag2^sm^* MuSCs using PolyJet In Vitro DNA Transfection Reagent (SL100688) for 3 hours. The *pcDNA3-JAG2* was also transfected to WT MuECs. For the generation of the stable cell lines, the medium was replaced with myoblast growth medium, and geneticin (G418, 300 μg/mL; 10131035, Gibco) was added for the selection of transformant cells, which were used for cell growth and differentiation assays.

### Western blotting.

Protein extracts of MuSC culture were used for Western blotting. Protein concentration was determined by the Micro BCA Protein Assay Reagent kit (Thermo Fisher Scientific). HES1 was detected by Western blotting with anti-HES1 antibody (C23D2) followed by anti–rabbit IgG HRP (31460, Cell Signaling Technology). To verify equal loading proteins, the same blots were stripped and reprobed with anti-GAPDH HRP conjugated (3683, Cell Signaling Technology) as a cytosolic marker. The reaction was developed using SuperSignal West Femto chemiluminescent substrate (PI37074, Thermo Fisher Scientific) in accordance with the manufacturer’s instructions.

### PLA.

Cells were inoculated into 3 cm culture dishes. The following day, cells were fixed with 4% PFA, followed by permeabilization for 5 minutes using PBS + 0.2% Triton X-100. After rinsing, the PLA was carried out using a DuoLink in situ PLA kit (DUO92101, MilliporeSigma). Following incubation with primary antibodies (anti-JAG2 and anti-NOTCH1, anti-NOTCH2, or anti-NOTCH3 antibodies; Supplemental Table 7), 2 distinct PLA probes (anti-rabbit minus and anti-mouse plus) were combined and incubated on the dishes for 1 hour. Microscopy images were captured as described above.

### Coculture.

Coculture was performed by plating a monolayer of MuECs overlaid with MuSCs and culturing them in low-serum media to induce myogenic differentiation and reserve cell induction for 5 days (6, 14, 15). *Jag2* siRNAs (sc-39673, Santa Cruz Biotechnology) and control scramble siRNA-A (sc-37007, Santa Cruz Biotechnology) were transfected in MuECs by Polyjet (11668019, Thermo Fisher Scientific) before coculture. *Hes1-467-Luc* was transfected to MuSCs prior to coculture experiments. A γ-secretase inhibitor, 10 μM DAPT (D5942, Sigma-Aldrich), was used to block NOTCH signaling. Following cocultures, anti-PAX7 (DSHB) and anti-MYOD (C-20, Santa Cruz Biotechnology) antibodies followed by secondary anti-mouse–Alexa Fluor 488 (A-11029) and anti-rabbit–Alexa Fluor 594 (A-21207, Thermo Fisher Scientific) antibodies were used to detect PAX7^+^MYOD^–^ self-renewing reserve cell populations (Supplemental Table 7). DAPI was used for nuclear staining.

### Grip strength test.

A forelimb grip strength test was performed (8). Mice were gently pulled by the tail after forelimb grasping a metal bar attached to a force transducer (Grip Strength Meter, 1027CSM-D52, Columbus Instruments). Grip strength tests were performed by the same blinded examiner. Five consecutive grip strength tests were recorded, and then the mice were returned to the cage for a resting period of 20 minutes. Then, 3 series of pulls were performed, each followed by a 20-minute resting period. The average of the 3 highest values out of the 15 values collected was normalized to the body weight for comparison.

### Treadmill running.

The Exer-3/6 Treadmill (Columbus Instruments) was used for treadmill running tests (8). For acclimation, mice in each lane ran on a treadmill for 5 minutes at 10 m/min on a 0% uphill grade daily for 3 days. Then, the mice ran on a treadmill with a 10% uphill grade, starting at 10 m/min for 5 minutes. Then, every 2 minutes, the speed was increased by 2 m/min until exhaustion, defined as the mice’s inability to remain on the treadmill. The running time and distance were recorded.

### Rotarod test.

Mice were trained on the rotarod (0890M-D54 Rotamex-5, Columbus Instruments) for 2 days before data collection (8). During each trial, mice were placed on the rod at 10 rpm for 60 seconds, and the rod accelerated from 10 to 30 rpm at 30-second intervals. The total maximum testing time was 240 seconds. Each trial was performed twice daily at 2-hour intervals for 3 consecutive days. The latency to fall was recorded, and the most prolonged latency was used for analysis.

### Transcriptome (RNA-seq) analysis.

3 WT and 4 *Jag2^sm^* homozygous RNA samples were extracted from MuSCs isolated from hind limb muscles. The RNA samples underwent Tapestation analysis (Agilent) to ensure RNA quality for long-read sequencing. 1 μg of each RNA sample was used as input for cDNA synthesis library prep following the Ligation Sequencing V14 direct cDNA sequencing (SQK-LSK114) protocol from Oxford Nanopore Technologies until elution at the cDNA repair and end-prep step. The cDNA was eluted in 23.5 μL water. Following cDNA end prep, barcodes were ligated so that the cDNA could be pooled. 22.5 μL end-prepped cDNA, 2.5 μL Native Barcode (NB05-11 from SQK-NBD114.24), and 25 μL Blunt/TA Ligase Master Mix were combined, following the Direct cDNA Sequencing native barcoding (SQK-DCS109 with EXP-NBD104 and EXP-NBD114) protocol. 5 μL EDTA was used to inactivate the ligation reaction after 20 minutes of incubation. Half of each barcoded cDNA sample was pooled into 1 library, and the remaining half was pooled into a second library. The rest of the library prep and loading followed the protocol for the Ligation Sequencing Amplicons, Native Barcoding Kit 24 V14 (SQK-NBD114.24). The barcoded pooled libraries were loaded on 2 different PromethION flow cells (FLO-PRO114M). Sequencing was performed on a P2-solo (Oxford Nanopore Technologies) for approximately 23 hours, after data acquisition had plateaued. The raw data were base called using dorado/0.5.3 (Oxford Nanopore Technologies; available at https://github.com/nanoporetech/dorado/releases/tag/v0.5.3) with the --min-qscore 7 --kit-name ‘SQK-NBD114-24’ --no-trim flags, and the data were assembled to the GRCm39 mouse genome. The resulting BAM file was demultiplexed by barcode using dorado demux using the --emit-fastq flag. The resulting FASTQ files from corresponding barcodes of the 2 different libraries were concatenated together, and then transcripts from each sample were counted using minimap2/2.17 htseq. The transcript counts were input into DESeq2 using R 4.3.0 using default analysis parameters, and genes with significantly different expression levels (adjusted *P* value < 0.05) were recorded. Genes with expression (baseMean < 10) were filtered out of gene enrichment analysis, but the results from the whole transcriptome were saved. GO analysis was performed using Metascape v3.5.20250101 (68). Gene information was obtained from the NCBI database (https://www.ncbi.nlm.nih.gov/gene).

### Transgenic Drosophila generation.

Human reference *JAG2* and variants were cloned from the *pcDNA3* constructs into the *pUASTattB* vector using the EcoRI and XbaI sites for *Drosophila* expression via the in-fusion cloning method (Takara). The resulting plasmids were sequenced and verified (Eurofins Genomics). Transgenic animals were generated by Bestgene through φC31 integrase–mediated transgenesis on the second chromosome landing site, attP40.

### Drosophila husbandry.

All *Drosophila* lines were maintained with standard Bloomington food at 25°C with 70% humidity and a 12-hour-light/12-hour-dark cycle. All experiments were conducted at 27°C to enhance transgene expression efficiency.

### Drosophila behavioral assays.

For a flight assay, 25 flies from each group were collected and aged for 4 days. They were funneled into a 500 mL glass cylinder. The distribution of flies in the cylinder was recorded by a document camera (IPEVO), and the average scores from 5 independent experiments were calculated. For a negative geotaxis assay, 25 flies from each group were collected and aged for 4 days. Flies were tapped down by gently striking the vials on the surface of a table. Their climbing behaviors were recorded by a document camera (IPEVO) for 30 seconds. Movie clips were exported and analyzed by ImageJ.

### Statistics.

Statistical analysis was performed using GraphPad Prism 10. For comparison between 2 groups, an unpaired 2-tailed Student’s *t* test was used. For comparison between multiple groups, 1-way ANOVA followed by Bonferroni’s post hoc test was used with multiple comparisons to the control. Distributions were compared using a χ^2^ test. Graphing of the data was performed using Prism 10. All values are presented as mean ± SEM unless noted otherwise. *P* values of less than 0.05 were considered significant.

### Study approval.

All protocols (2204–39969A) were approved by the IACUC of the University of Minnesota and complied with NIH guidelines for the use of animals in research.

### Data availability.

All data associated with this study are included in the article or the supplemental material, with the exception of the transcriptome data, which have been uploaded to the Gene Expression Omnibus with accession number GSE327287. Supporting datasets, including quantified values and analysis outputs, are provided in the Supporting Data Values file.

## Author contributions

MT, NCK, HRL, MG, JT, NMW, QM, YA, and AA conducted the experiments, collected the data, and analyzed the results. ID provided support and advice regarding the study design and experimental methods. MT, NCK, PBK, and AA conceived and designed the study and drafted the manuscript. The order of co–first authors and co–corresponding authors was determined through mutual discussion and agreement.

## Conflict of interest

The authors have declared that no conflict of interest exists.

## Funding support

This work is the result of NIH funding, in whole or in part, and is subject to the NIH Public Access Policy. Through acceptance of this federal funding, the NIH has been given a right to make the work publicly available in PubMed Central.

- NIH grants 1R21AR079033 and 1R21AR078400.
- Department of Defense award (HT9425-23-1-0461 to AA).
- Muscular Dystrophy Association research grant (1297954 to AA).
- Greg Marzolf Jr. Research Foundation to AA.

## Supplementary Material

Supplemental data

Unedited blot and gel images

Supplemental table 1

Supplemental table 2

Supplemental table 3

Supplemental table 4

Supplemental table 5

Supplemental table 6

Supplemental table 7

Supporting data values

## Figures and Tables

**Table 1:**
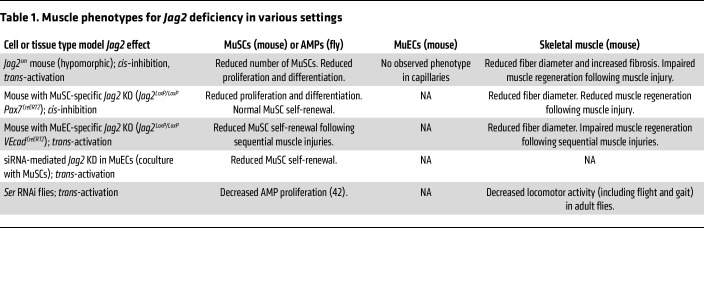
Muscle phenotypes for *Jag2* deficiency in various settings

## Version 1 05/19/2026

In-Press Preview

## Version 2 07/01/2026

Electronic publication
