# Supplementary material for: Deficiency of muscular dystrophy–related gene JAG2 causes NOTCH signaling dysfunction in muscle stem cells
Source: J Clin Invest. 2026 May 19;136(13):e198639. doi: 10.1172/JCI198639 (PMC13318124; doi:10.1172/JCI198639)
Supplement: Supplemental data [file jci-136-198639-s089.pdf]

Figure S1

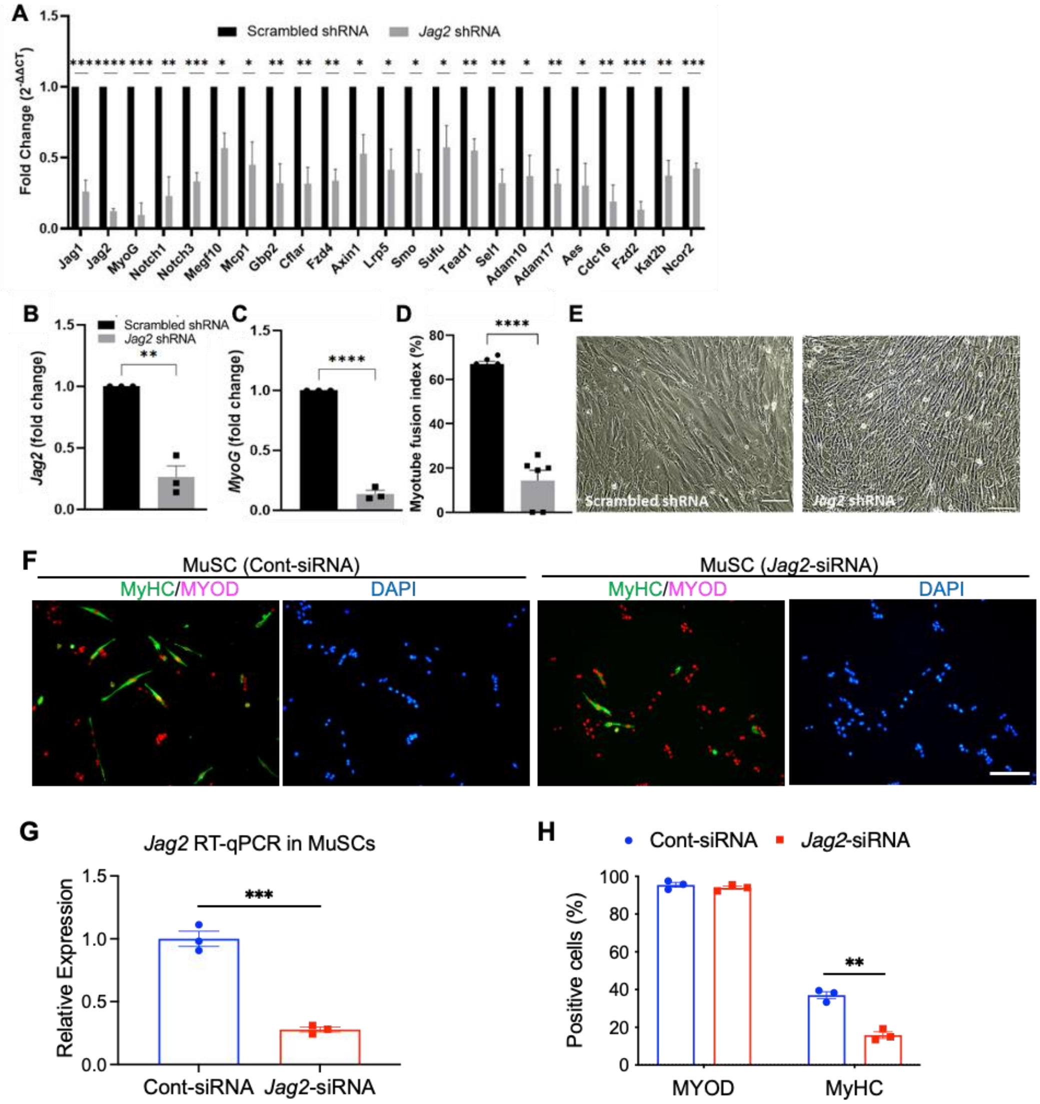

Figure S1. Impaired myogenic differentiation and Notch signaling in *JAG2* deficient-myoblasts. (A) *Jag2* shRNA and scrambled shRNA C2C12 myoblasts were differentiated for 7 days. RT-qPCR was performed on isolated RNA with an array plate containing probes for 93 Notch signaling and myogenesis genes, along with 3 control genes. The 23 significantly downregulated genes are shown in the graph, including *MyoG*, *Notch1*, and *Notch3*. No genes were significantly upregulated. Transcript levels were normalized to *Gapdh*. (B-D) The down-regulated genes were confirmed by RT-qPCR. (E) Phase contrast images of day 7-differentiated *Jag2* knockdown cells show a lack of myotubes compared to scrambled shRNA cells. (F) WT MuSCs were transfected with control or *Jag2* siRNA, cultured in differentiation medium for 1 day and stained with MYOD (red) and MyHC(+) for myogenic differentiation (green). DAPI stained all nuclei (blue). (G) *Jag2* expression was assessed by RT-qPCR following transfection with control or *Jag2*-siRNA. Transcript levels were normalized to *18S*. (H) Number of nuclei harboring MyHC- or MYOD-positive cells on day 1 of differentiation in control cells and *Jag2*-siRNA-treated cells. Scale bars, 100 µm. Student's t-tests showed *p<0.05, **p<0.01, ***p<0.001 and ****p<0.0001. Error bars show SEM [(A, B, C, G, H) n=3, and (D) n=5 biological replicates].

Figure S2

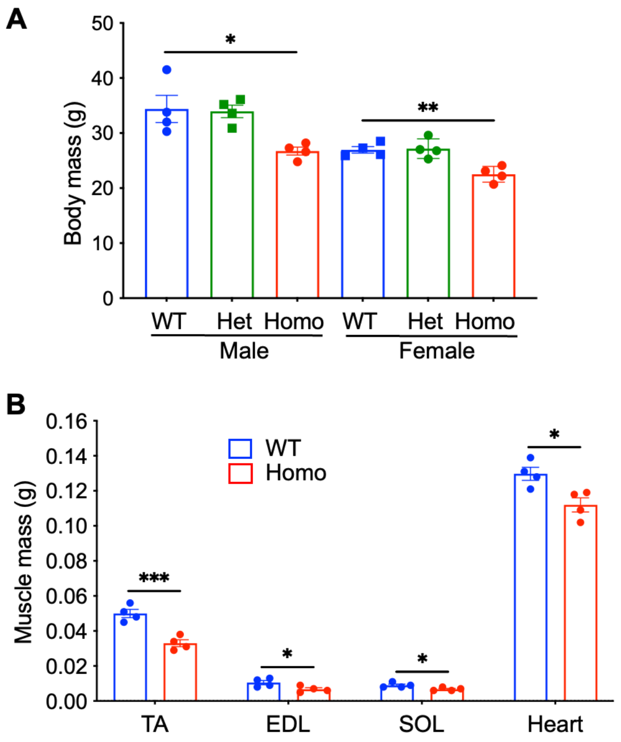

Figure S2. Reduced body and muscle mass in homozygous *Jag2*^{sm} mice. Three-month-old *Jag2*^{sm} homozygous male and female mice showed reduced body mass vs. *Jag2*^{sm} WT and/or heterozygous mice. Three-month-old *Jag2*^{sm} homozygous male tibialis anterior (TA), extensor digitorum longus (EDL), soleus (SOL) muscle, and heart showed reduced muscle mass vs. *Jag2*^{sm} WT mice. Student's t-tests showed *$p<0.05$, **$p<0.01$ and ***$p<0.001$. Error bars show SEM (n=4 biological replicates).

Figure S3

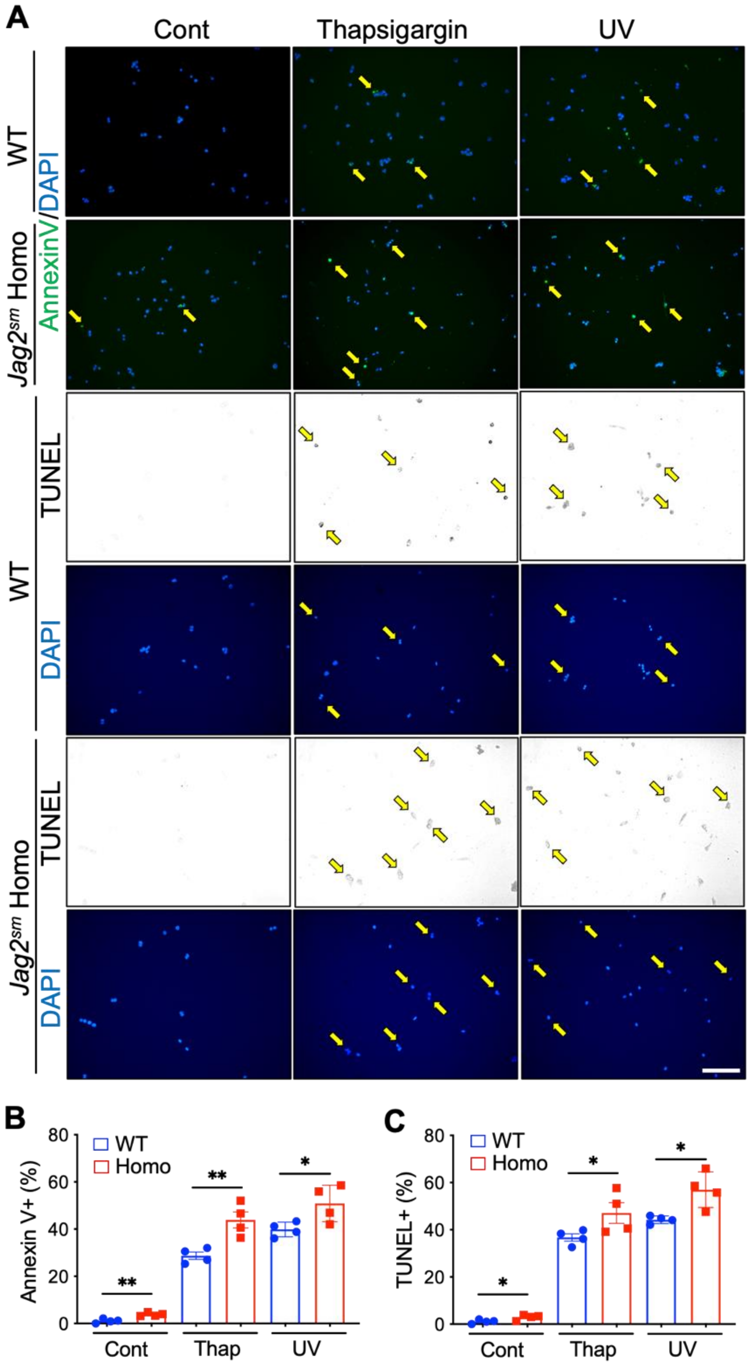

Figure S3. *Jag2*^{sm} MuSCs show increased apoptotic cell death. MuSCs isolated from WT and homozygous *Jag2*^{sm} mice were used for detection of apoptosis [arrows, Annexin V(+) green-stained cells or TUNEL(+) gray-stained cells]. Twenty-four hours following treatment with thapsigargin or UV exposure. DAPI stained all nuclei (blue). Scale bars, 100 μm. Student's t-tests showed *p<0.05 and **p<0.01. Error bars show SEM (n=4 biological replicates).

Figure S4

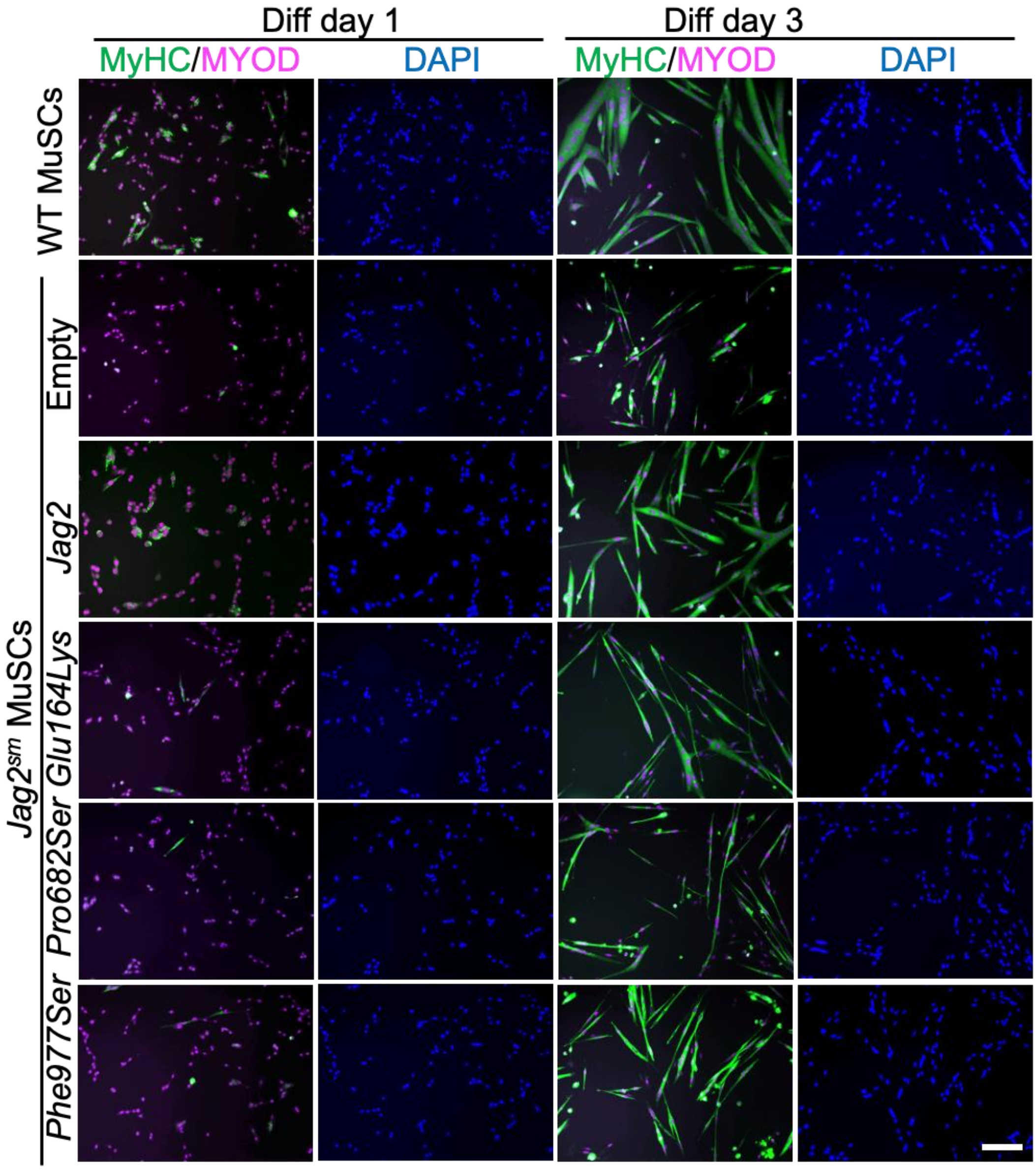

Figure S4. Overexpression of *JAG2* rescues differentiation defects in *Jag2^{sm}* MuSCs. MuSCs isolated from WT and homozygous *Jag2^{sm}* mice were used for overexpression of human reference *JAG2* (*Jag2^{WT}*) and human *JAG2* harboring three patient variants (*p.Glu164Lys*, *p.Pro682Ser*, and *p.Phe977Ser*). Overexpression of *Jag2^{WT} but not JAG2* variants increased expression and MyHC(+) myogenic differentiation (green) and rescued myogenic differentiation defects in *Jag2^{sm}* MuSCs. MYOD expression was shown in nuclei (purple). DAPI stained all nuclei (blue). Scale bars, 100 µm.

Figure S5

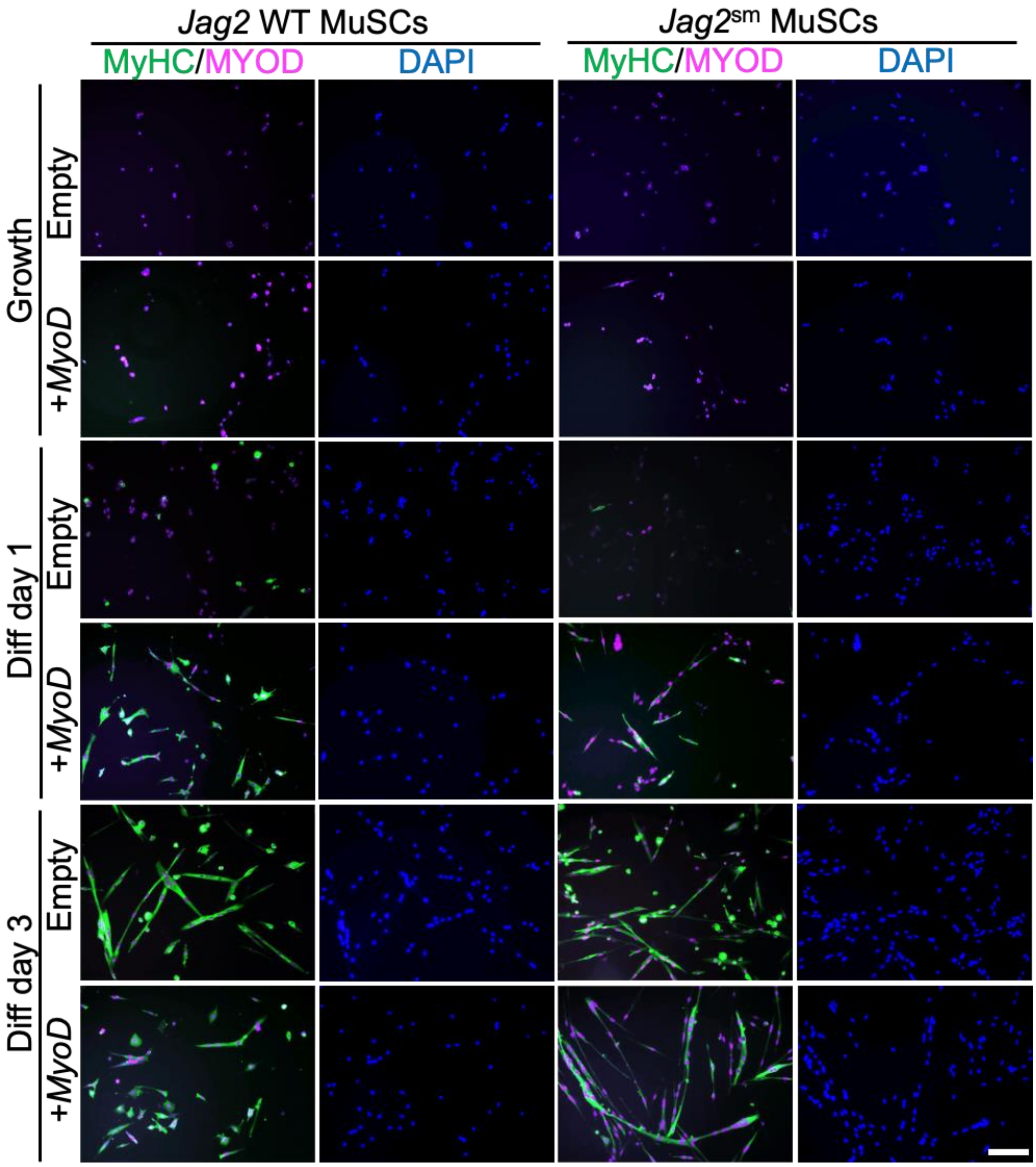

Figure S5. *MyoD* rescue myogenic differentiation in *Jag2^{sm}* MuSCs and direct binding of Jag2 to Notch1-3. MuSCs isolated from WT and homozygous *Jag2^{sm}* mice were used for overexpression of *MyoD*. Overexpression of *MyoD* increased MYOD (purple) expression and MyHC(+) myogenic differentiation (green) and rescued myogenic differentiation defects in *Jag2^{sm}* MuSCs. DAPI stained all nuclei (blue). Scale bars, 100 μm.

Figure S6

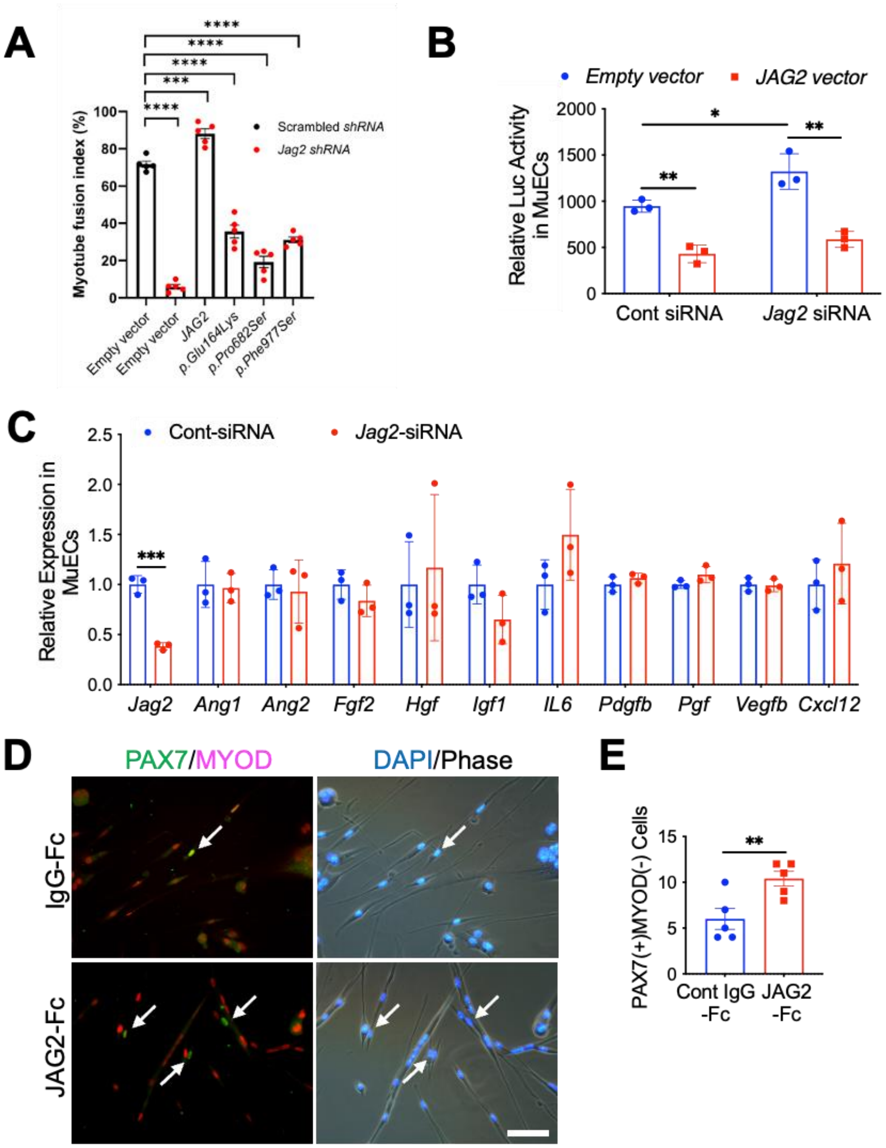

Figure S6. **Effects of *Jag2* KD and overexpression in MuECs and MuSCs.** (A) *Jag2* shRNA cells with stable overexpression of empty vector, *JAG2* and three patient variants were differentiated for 6 days. Scrambled shRNA cells was used for the control. Following MyHC staining, myotube fusion index was quantified. *Jag2* knockdown reduced the myotube fusion but overexpression of *JAG2* but not the variants rescued this defect. (B) MuECs were stably transfected with empty vector or *JAG2* expression vector. In MuECs, overexpression of *JAG2* decreased *Hes1-467-Luc* activity compared with the empty vector following transfection. Transfection with siRNA for *Jag2* increased *Hes1-467-Luc* activity compared with control scramble siRNA. (C) Gene expression of *Jag2* and secreted molecules involved in MuSC proliferation and differentiation were compared between MuECs with control scramble siRNA and siRNA for *Jag2.* (D, E) PAX7(+)MYOD(-) reserve cells (arrows) were assessed in MuSCs exposed to control-IgG-Fc or the extracellular JAG2-Fc for 5 days in differentiation medium. DAPI stained all nuclei (blue). Scale bar, 100 µm. One-way Anova followed by the Bonferroni post hoc tests showed ***p<0.001 and ****p<0.0001. Student's t-tests showed *p<0.05, **p<0.01 and ***p<0.001. Error bars show SEM [(A, E) n=5, and (B, C) n=3 biological replicates].

Figure S7

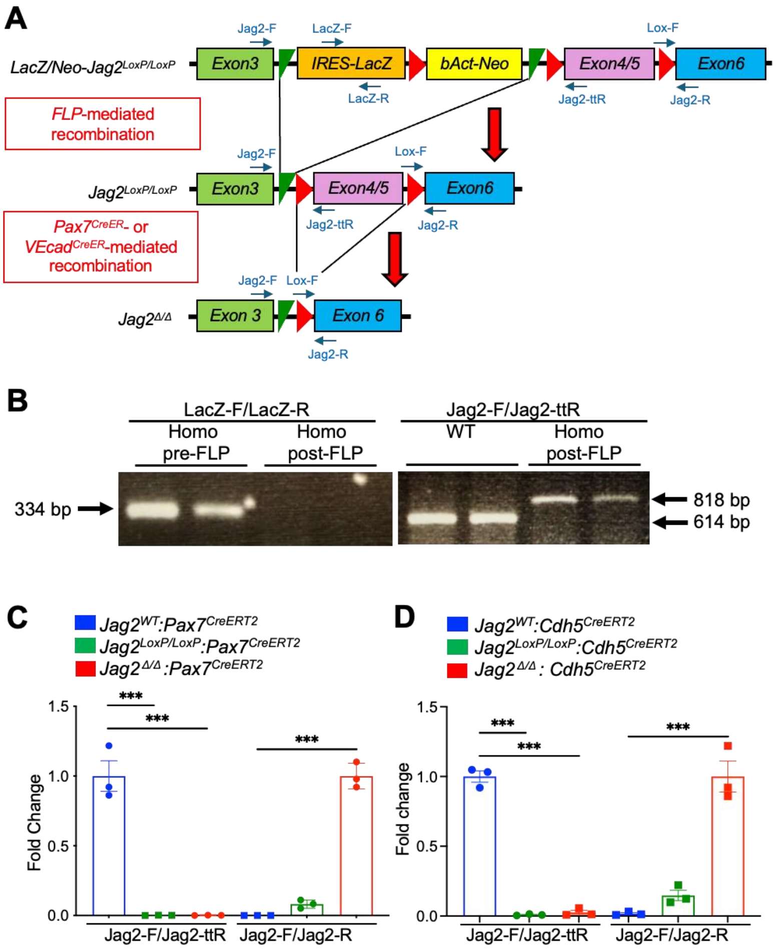

Figure S7. Confirmation of conditional deletion of *Jag2*. (A) Schematic genomic structure of *Jag2^{floxed}* locus for conditional *Jag2* mutant (*LacZ/Neo-Jag2$^{LoxP/LoxP}$*) mice. Following breeding with *FLP* mice, Flippase-Frt sites (green triangles)-mediated *IRES-LacZ/bAct-Neo* cassette deletion occurred. Following breeding with *Pax7^{CreER}* or *VEcad^{CreER}* mice and TMX-treatment, *CreER-LoxP* sites (red triangles)-mediated *Exons 4/5* deletion occurred. (B) Genomic PCR was performed to detect the *LacZ* gene in pre-*FLP* but not in post-*FLP* mice in homozygous *LacZ/Neo-Jag2$^{LoxP/LoxP}$* mice using *Lac-F/LacZ-R* primers. Following breeding with *FLP* mice, an 818 bp band appeared in homozygous *LacZ/Neo-Jag2$^{LoxP/LoxP}$* mice while WT mice showed a 614 bp band using *Jag2-F/Jag2-ttR* primers. (C) MuSCs isolated from *Jag2$^{LoxP/LoxP}$:Pax7^{CreER}* (without TMX) or *Jag2$^{\Delta/\Delta}$:Pax7^{CreER}* (with TMX) mice showed no DNA amplification by qPCR using *Jag2-F/Jag2-ttR* primers, while MuSCs from *Jag2^{WT}:Pax7^{CreER}* mice showed the DNA amplification. Following treatment with TMX, qPCR using *Jag2-F/Jag2-R* primers amplified the DNA in MuSCs from *Jag2$^{\Delta/\Delta}$:Pax7^{CreER}* mice, while *Jag2^{WT}:Pax7^{CreER}* or *Jag2$^{LoxP/LoxP}$:Pax7^{CreER}* mice showed no DNA amplification. (D) MuECs from *Jag2$^{LoxP/LoxP}$:VEcad^{CreER}* (without TMX) or *Jag2$^{\Delta/\Delta}$:VEcad^{CreER}* (with TMX) mice showed no DNA amplification by qPCR using *Jag2-F/Jag2-ttR* primers, while *Jag2^{WT}:VEcad^{CreER}* showed DNA amplification. Following treatment with TMX, qPCR using *Jag2-F/Jag2-R* primers amplified DNA in MuECs from *Jag2$^{\Delta/\Delta}$:VEcad^{CreER}* mice but not in *Jag2^{WT}:VEcad^{CreER}* or *Jag2$^{LoxP/LoxP}$:VEcad^{CreER}* mice. One-way Anova followed by the Bonferroni post hoc tests showed ***$p<0.001$. Error bars show SEM (n=3 biological replicates).

Figure S8

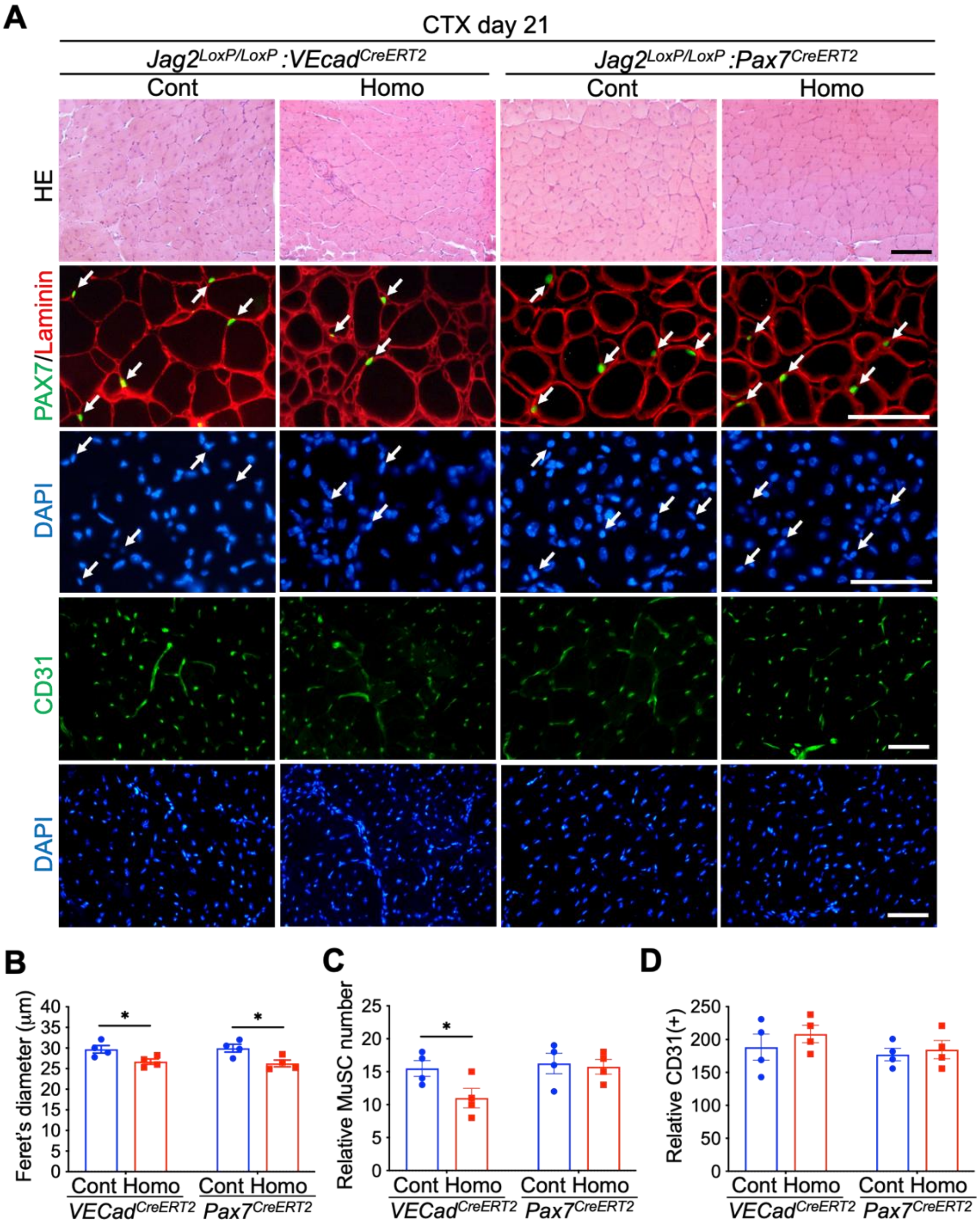

Figure S8. Reduced self-renewal in *Jag2$^{LoxP/LoxP}$:VEcad^{CreERT2}* and reduced regeneration in *Jag2$^{LoxP/LoxP}$:Pax7^{CreERT2}* mice. (A) Following TMX injection, CTX injections into the TA were performed in *Jag2$^{LoxP/LoxP}$:VEcad^{CreERT2}* and *Jag2$^{LoxP/LoxP}$:Pax7^{CreERT2}* mice. H&E, anti-PAX7 (green, arrows)/Laminin (red) and anti-CD31 (green) antibody staining were performed by 21 days following CTX injection. DAPI stained all nuclei (blue). (B) Feret's diameters of TA muscle fibers, (C) anti-PAX7(+) MuSCs, and (D) CD31(+) capillaries were counted. Scale bars, 100 µm. Student's t-tests showed *$p<0.05$. Error bars show SEM (n=4 biological replicates).
