# Supplementary figures and images for: Deficiency of muscular dystrophy–related gene JAG2 causes NOTCH signaling dysfunction in muscle stem cells
Source: J Clin Invest. 2026 May 19;136(13):e198639. doi: 10.1172/JCI198639 (PMC13318124; doi:10.1172/JCI198639)

# Unedited blotting Images for Figure 6I and 6J

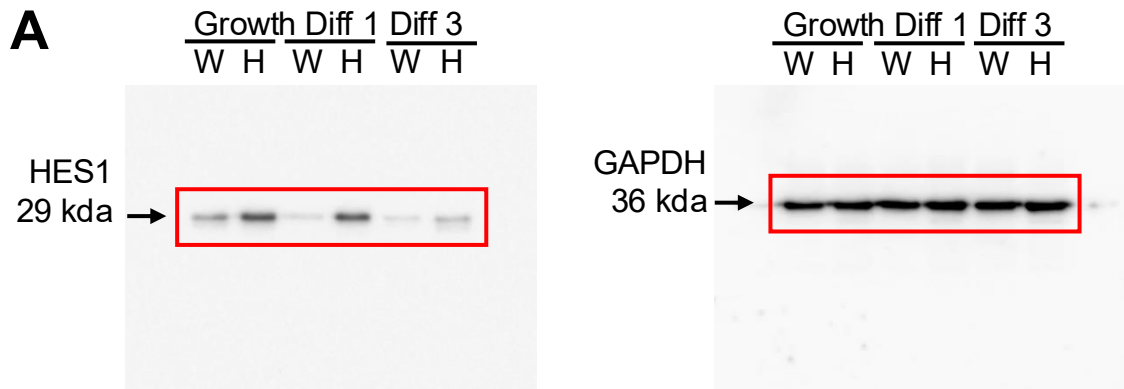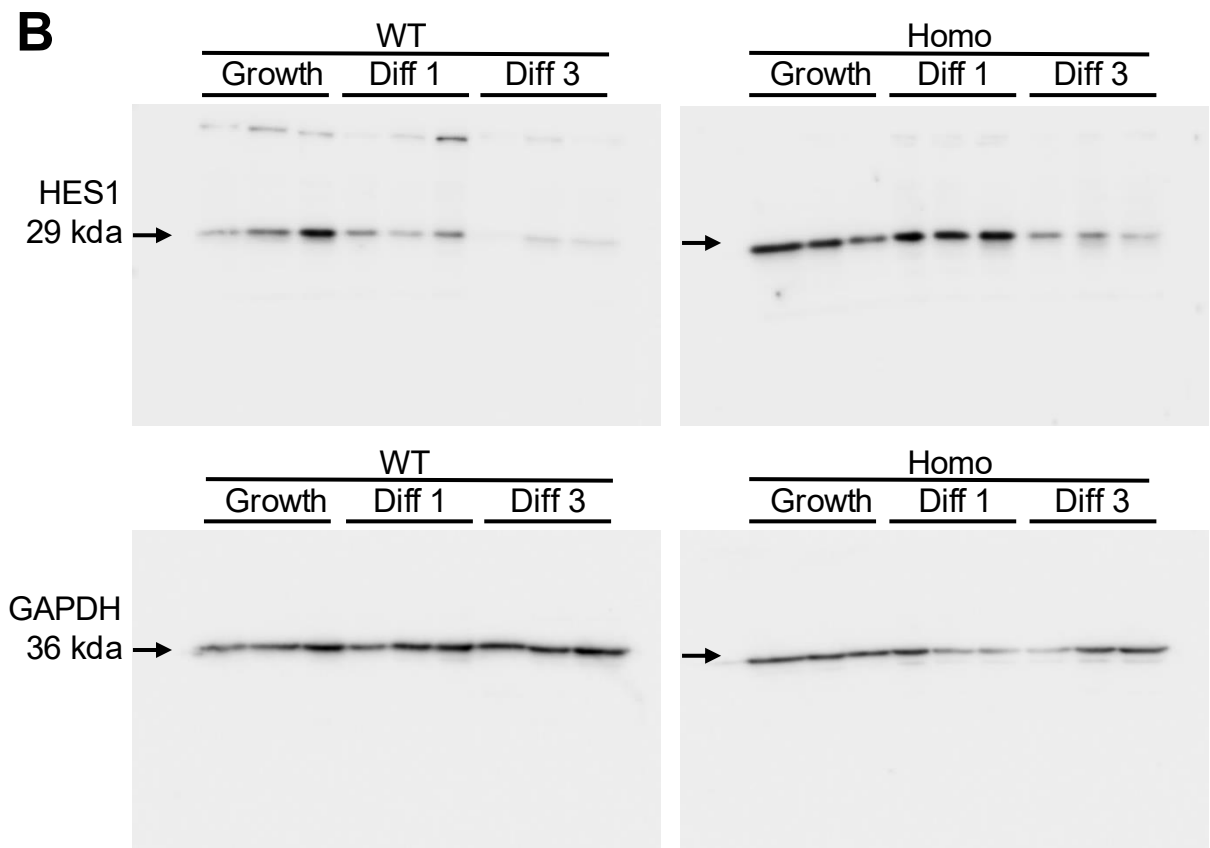

# Unedited Gel Images for Supplemental Figure 7B

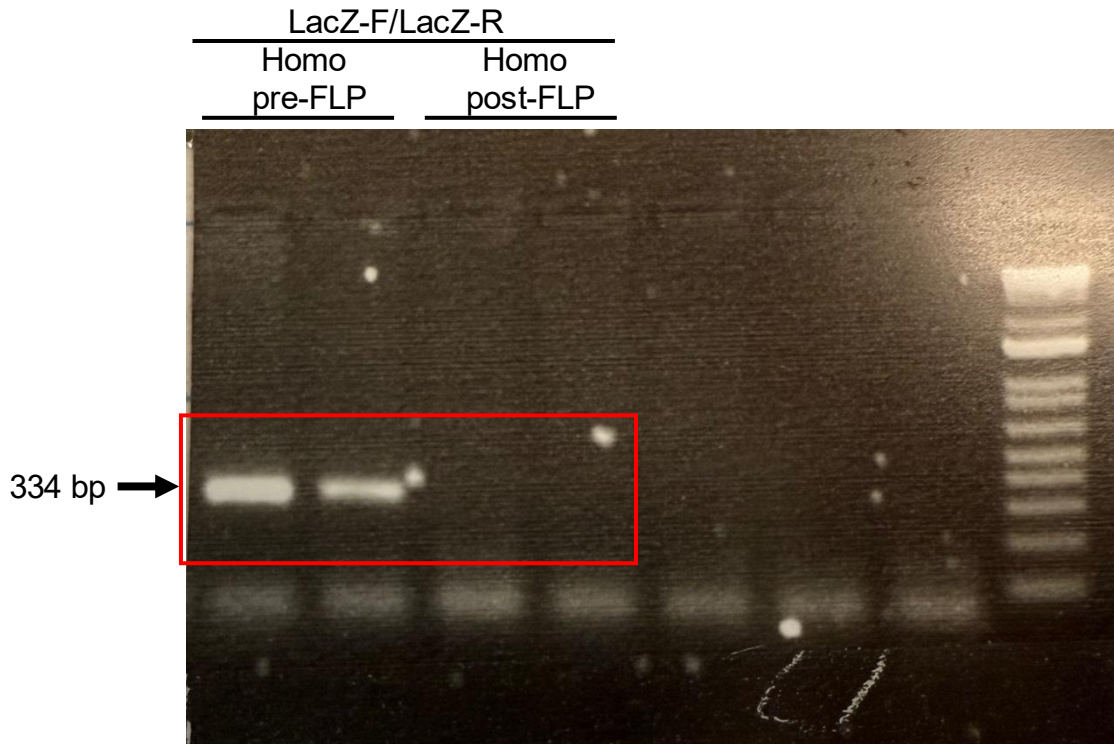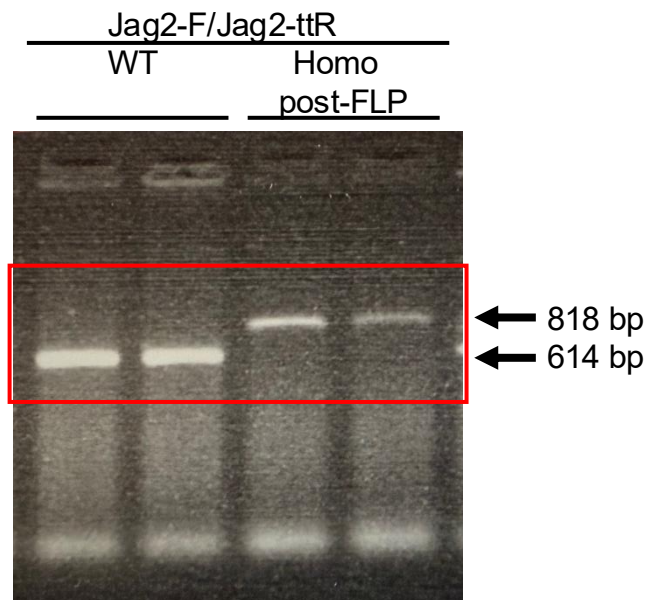

Supplement: Unedited blot and gel images [file jci-136-198639-s090.pdf]
